# *Gluconacetobacter diazotrophicus* AZ0019 requires functional *nifD* gene for optimal plant growth promotion in tomato plants

**DOI:** 10.3389/fpls.2024.1469676

**Published:** 2024-11-22

**Authors:** Michele Pallucchini, Martina Franchini, Enas M. El-Ballat, Nathalie Narraidoo, Benjamin Pointer-Gleadhill, Matthew J. Palframan, Christopher J. Hayes, David Dent, Edward C. Cocking, Michele Perazzolli, Rupert G. Fray, Phil J. Hill

**Affiliations:** ^1^ The University of Nottingham, School of Biosciences, Plant Sciences Division, Sutton Bonington, Leicestershire, United Kingdom; ^2^ Azotic Technologies Ltd., Dunnington, United Kingdom; ^3^ Research and Innovation Centre, Fondazione Edmund Mach, San Michele all’Adige, Italy; ^4^ Botany Department, Faculty of Science, Tanta University, Tanta, Egypt; ^5^ The University of Nottingham, School of Chemistry, Nottingham, United Kingdom; ^6^ University of Wolverhampton, School of Pharmacy, Wolverhampton, United Kingdom; ^7^ The Sustainable Nitrogen Foundation, Cutbush House, Saham Toney, United Kingdom; ^8^ Centre Agriculture Food Environment (C3A), University of Trento, San Michele all’Adige, Italy; ^9^ The University of Nottingham, School of Biosciences, Division Microbiology, Brewing and Biotechnology, Sutton Bonington, Leicestershire, United Kingdom

**Keywords:** nitrogen fixation, plant growth promotion, tomato, hydroponics, *Gluconacetobacter diazotrophicus*

## Abstract

*Gluconacetobacter diazotrophicus* is a nitrogen fixing bacterium able to colonise a wide range of host plants and is marketed as a biofertiliser due to its ability to promote plant growth. This study aims to investigate how biological nitrogen fixation (BNF) competency affects the growth promotion of inoculated tomato plants and to describe the colonisation mechanism of this bacterium in dicot systems. A nitrogen fixation impaired mutant (Gd *nifD^-^*) was produced by disrupting the *nifD* gene, which encodes the nitrogenase Mo-Fe subunit, in order to assess its plant growth promotion (PGP) capability in comparison to *G. diazotrophicus* wild type strain (Gd WT). Furthermore, tagged strains were employed to monitor the colonisation process through qPCR analyses and fluorescence microscopy. Following a preliminary glass house trial, Gd WT or Gd *nifD^-^* were applied to hydroponically grown tomato plants under nitrogen-replete and nitrogen-limiting conditions. Bacteria reisolation data and plant growth parameters including height, fresh weight, and chlorophyll content were assessed 15 days post inoculation (dpi). Gd WT significantly enhanced plant height, fresh weight, and chlorophyll content in both nitrogen conditions, while Gd *nifD^-^* showed a reduced PGP effect, particularly in terms of chlorophyll content. Both strains colonised plants at similar levels, suggesting that the growth advantages were linked to BNF capacity rather than colonisation differences. These findings indicate that a functional *nifD* gene is a fundamental requirement for optimal plant growth promotion by *G. diazotrophicus*.

## Introduction

Nitrogen (N) is one of the most important nutrients for plants’ development, being a primary constituent of nucleotides, proteins, and chlorophyll. The limited natural N supply is a restriction to crop yield; therefore, crop productivity relies heavily on N fertilisation. The use of chemical N fertilisers has brought positive effects on the cropping systems in terms of yield and productivity but came with huge energy costs as well as environmental damage, such as the contribution to greenhouse gas emissions during the chemical N fertiliser production processes (Vitousek et al., 1997; Erisman et al., 2008). Agricultural systems that use available nitrogen more efficiently, or which utilise localised biological nitrogen fixation (BNF) would allow reduced chemical fertiliser inputs. BNF implies the reduction of atmospheric dinitrogen (N_2_) to ammonia by means of prokaryotes. This mechanism has been extensively studied in the diversified population of N-fixing bacteria (diazotrophs) (Reed et al., 2011). Particular interest has been drawn by BNF in bacteria that live associated with plants, although they only represent a portion of diazotrophs, for their potential in agricultural applications (Imran et al., 2021; Xu and Wang, 2023). While symbiotic N-fixing systems involving rhizobia and the formation of nodules in legumes are widely studied and well-characterised (Manchanda and Garg, 2007), many diazotrophs do not rely on this process. Among these non-nodulating bacteria, some N-fixing cyanobacteria, such as *Nostoc* sp., have been found to colonise different plant families (e.g. *Gunneraceae*, liverwort, hornwort, *Azolla* and *Cycadaceae*) (Santi et al., 2013). Other diazotrophs such as *Herbaspirillum*, *Azospirillum* spp. and *Azoarcus* spp. are routinely found associating with a wide range of plants, though their contribution to the nitrogen cycle is not as well characterised (Santi et al., 2013). While fully capturing the complexity and relevance of interactions between non-nodulating diazotrophs and their hosts is challenging, recent studies highlight their importance and even suggest the presence of specialised diazotroph-harbouring organelles (Coale et al., 2024).

*Gluconacetobacter diazotrophicus* is a non-nodulating, N-fixing, Gram-negative acetic acid bacterium (Dent, 2018) first isolated in 1988 from sugarcane plants in Brazil (Cavalcante and Dobereiner, 1988). *G. diazotrophicus* was classified as an endophyte since it is not found as a free-living soil bacterium but has been isolated in the rhizosphere closely associated with roots, which are likely to provide carbon and other nutrients that are fundamental for its growth (Sevilla et al., 2001). It was shown to reside mainly in the apoplast of sugarcane plants, in both roots and stems (Cavalcante and Dobereiner, 1988) and to be capable of xylem colonisation (James et al., 2001; Dong et al., 1997).

*G. diazotrophicus* was also shown to have the ability to synthesise a range of phytohormones, including Indole-3-acetic acid (Fuentes-Ramirez et al., 1993) and gibberellins A1 and A3 (Bastián et al., 1998).

Besides sugarcane, *G. diazotrophicus* has been reported to be associated with 19 plant species representing 15 plant families (Dent, 2018), including sweet potato (Paula et al., 1991), pineapple (Tapia-Hernández et al., 2000), coffee (Jimenez-Salgado et al., 1997), tea, mango, banana, rice (Muthukumarasamy et al., 2002b), corn (Tian et al., 2009), sorghum (Paula et al., 1991), and tomato (Restrepo et al., 2017).

Among the suitable hosts for *G. diazotrophicus*, tomato was chosen as a case of study for this work. Tomatoes, one of the most economically valuable crops worldwide, represent the second most consumed vegetable globally (FAOSTAT, 2024). Previous works have proved *G. diazotrophicus* to be able to significantly increase the number and weight of tomato fruits produced in inoculated plants grown in soil (Luna et al., 2012) and to improve root and aerial biomass production of inoculated seedlings grown on MS agar (Botta et al., 2013). Our study focused on the evaluation of the effect of *G. diazotrophicus* inoculation on tomatoes grown in a hydroponic system. The aim of this work was to describe the colonisation process and assess the relevance of the nitrogen fixation capability of *G. diazotrophicus* to the plant growth promoting effect on tomato plants. This was achieved by comparing the effect of a nitrogen fixation impaired *G. diazotrophicus* mutant (Gd *nifD^-^*) on tomato against the wild type bacterium (Gd WT). Additionally, this study provides a comprehensive account of the bacterium colonisation strategy and demonstrates its capability of cytoplasmic invasion in protoplasts prepared from *G. diazotrophicus* inoculated leaves.

## Materials and methods

### Bacteria growth conditions

All the *G. diazotrophicus* strains (**Supplementary Table S1**) were routinely grown in ATGUS medium [glucose 2.7 g L^-1^; mannitol 1.8 g L^-1^; yeast extract 2.7 g L^-1^; K_2_HPO_4_ 4.8 g L^-1^; KH_2_PO_4_ 0.65 g L^-1^; MES hydrate 4.4 g L^-1^; final pH 6.5 with acetic acid] (Cocking et al., 2006) at 28°C. For *nifD^-^* growth, kanamycin (50 µg mL^-1^) was added to the growth medium. *E. coli β2163* donor strains for biparental mating were grown in LB-Miller medium [yeast extract 5 g L^-1^, peptone 10 g L^-1^, NaCl 10 g L^-1^] at 37°C with the addition of the appropriate antibiotics and 0.06 g L^-1^ (0.3 mM) diaminopimelic acid (DAP). *G. diazotrophicus* cells were re-isolated from plant tissues on ace-LGIP medium (adapted from Sevilla et al., 1998) [sucrose 100 g L^-1^; yeast extract 0.025 g L^-1^; KH_2_PO_4_ 0.75 g L^-1^; CH_3_CO_2_K 0.35 g L^-1^; MgSO_4_·7H_2_O 0.02 g L^-1^; CaC1_2_·2H_2_O 0.02 g L^-1^; Na_2_MoO_4_·2H_2_O 0.002 g L^-1^; FeCI_2_.6 H_2_O 0.01 g L^-1^; bromothymol blue 0.5% solution in 0.2 N KOH 5 mL; agar 15 g L^-1^; final pH 4.0 with acetic acid] at 28°C.

### Bacterial strains

*Gluconacetobacter diazotrophicus* strain AZ0019 (Gd WT) was provided by Azotic Technologies Ltd, United Kingdom. This strain is derived from *G. diazotrophicus* UAP5541 (Caballero-Mellado and Martinez-Romero, 1994) and is maintained under the designation AZ0019 in the Azotic Technologies Ltd strain collection.

The nitrogen fixation impaired mutant strain (Gd *nifD^-^*) was obtained through biparental mating with *E.coli β2163* carrying the pSW23T suicide plasmid (Demarre et al., 2005) with the *nifD^-^* disruption cassette (**Supplementary Methods S1**, **Supplementary Figure S1**).

A *gfp*-tagged *G. diazotrophicus* strain was obtained by transformation through electroporation of a pBBR1MCS5-GFP [Cm^R^; Gent^R^] plasmid carrying a gfpmut3*-*cat* cassette (Andersen et al., 1998). Briefly, 100 µl of electrocompetent cells were transferred to a chilled 2 mm electroporation cuvette and subjected to a 1800 V pulse in a Gene Pulser apparatus (Bio-Rad). Positive clones were selected on ATGUS medium with appropriate antibiotics (**Supplementary Table S1**) and GFP screening by fluorescence microscopy. A *dsRed*-tagged *G. diazotrophicus* strain was obtained using the same protocol through electroporation with the pICH47751 plasmid harbouring a *dsRed-Express2* gene and a kanamycin resistance gene. The *gfp*-tagged strain was further transformed with the pRGS561 plasmid [Spe^R^; St^R^; Kan^R^] (Fuentes-Ramírez et al., 1999) carrying a constitutive GUS::NPTII cassette, via conjugation with the *E. coli β2163* donor strain. Double transformants were selected on ATGUS with appropriate antibiotics (**Supplementary Table S1**) and screened for both GFP fluorescence and blue staining after the addition of 100 µg mL^-1^ X-Gluc to ATGUS medium.

Plasmid extractions were performed with the GenElute Plasmid DNA Miniprep Kit (Sigma-Aldrich, Merck).

### Bacterial inocula preparation

Gd WT and Gd *nifD^-^* were streaked on solid ATGUS medium (with suitable antibiotics where required) from -80°C stocks. After three days of incubation at 28°C, a 20 mL liquid pre-inoculum was prepared starting from 3 colonies collected from plate cultures and incubated overnight at 28°C with agitation at 200 rpm.

The pre-inoculum was used to re-inoculate 500 mL of liquid ATGUS in a 1 L flask. Growth was carried out overnight at 28°C in agitation at 200 rpm. Bacteria were aliquoted in sterile 50 mL tubes and pelleted at 4000 *x* g (Thermo Scientific™ TX-400 4 x 400 mL Swinging Bucket Rotor), at 21°C for 10 minutes. Bacteria were washed and re-pelleted twice with sterile distilled water.

### Seed coating

After pelleting and washing from the ATGUS medium, bacterial cells were resuspended in a coating adjuvant [sucrose 30 g L^-1^; gum Arabic 3 g L^-1^; Tween 80 1 mL v/v] to reach a concentration of 10^9^ CFU mL^-1^ (confirmed by plate counts). Seeds of *Solanum lycopersicum* L. cv. MoneyMaker (Mole’s Seeds, UK; Just Seed, UK) were surface sterilised by soaking with 70% ethanol for 10 min followed by vigorous washing with sterile distilled water; subsequently, seeds were soaked in 5% sodium hypochlorite for 10 min and washed seven times with sterile distilled water. 0.4 g of *S. lycopersicum* L. cv. MoneyMaker seeds were soaked in ~5 ml of each of the bacterial suspension for 30 mins at room temperature. Mock-treated control seeds were soaked in the same volume of sterile, uninoculated coating adjuvant. After the 30 mins incubation, seeds were drained from the liquid and spread on a sterile Petri dish, in a sterile cabinet, until dry.

### Glasshouse growth conditions

*S. lycopersicum* L. cv. MoneyMaker seeds were sown into Levington M3 soil (premixed with 30 mg L^-1^ of T34 powder, Fargro) and incubated in a glasshouse with a 16-8 light-dark photoperiod at 24°C during the day and 20°C at night. One month after germination, plantlets were transferred from germination trays into 10 L pots filled with Klasmann Tray substrate mixed with Silver Sand (50:50) and routinely watered with N-free feeding (0N:36P:36K g L ^-1^). These plants were used in the preliminary assessment of growth promotion in soil following seed inoculation. Plants were kept in the glasshouse for 4 months. Throughout growth, side shooting was performed and biomass obtained was collected, oven dried and average dry weight per plant was calculated. Moreover, ripened fruits were collected, counted and weighed. Per-plant average weight and number of tomatoes produced are shown as an estimate of plant yield. Total plants biomass (above-ground) was also collected at the end of the experiment, oven dried and average per plant dry biomass was calculated.

### Tomato plant inoculation and growth conditions in hydroponics

Surface sterilised seeds were sown in sterile hydroponic boxes (10/15 seeds per box) filled with half strength (0.5x) modified Hoagland solution (NS; **Supplementary Table S3**). The N replete condition was set at 2 mM KNO_3_ (Jensen and Malter, 1995). Germination and growth were performed in a growth chamber (Conviron/Binder kbwf720, Bohemina, NY, USA) set at 80% humidity, 16 hours photoperiod, at a temperature of 25 ± 2°C. After seed germination, only plantlets exhibiting the same developmental stage and root length were kept, while outliers were discarded, and the same number of plants were maintained in each system. Ten days after seed germination, the washed and pelleted bacterial inocula were re-suspended in NS and inoculated in the nutrient solution of hydroponic systems at the final concentration of 10^7^ CFU mL^-1^ (OD_600_ = 0.3). After 72 hrs, the bacterial suspension was poured off from the hydroponic systems and replaced with fresh nutrient solution. Fifteen days after the bacterial inoculation, plant phenotypes were evaluated.

### Bacterial re-isolation from tomato plants

The colonisation level was assessed through the Most Probable Number (MPN) method (Cochran, 1950). Fifteen days after inoculation, inoculated plants and uninoculated controls were collected, and each plant was washed twice with 10 mM MgCl_2_ to remove loosely attached/non-adhering/non-interacting bacteria from the plants’ surface. Roots and shoots samples were then detached and separately ground in a mixer-mill disruptor (MM 400, Retsch, Haan, Germany) at 25 Hz for 1 min (or until completely macerated). Samples were then re-suspended in 200 µL of sterile water and carefully vortexed. Each suspension was serially diluted and 10 µL aliquots were plated in triplicate on ace-LGIP medium. After incubation at 28°C for three days, colony forming units (CFUs) of *G. diazotrophicus* per unit of plant fresh weight (CFUs g^-1^) were calculated. Colony PCR was performed on bacterial colonies employing strain specific primers 11 and 12 (**Supplementary Table S2**) to confirm bacterial identification after re-isolation from plants.

### RT-qPCR analyses

Tomato plantlets were collected from hydroponic systems inoculated with Gd WT or from uninoculated controls, in both zero N and N replete conditions, at 15 dpi. Roots and shoots were separated and processed as separate samples. After a quick wash with 10 mM MgCl_2_, roots or shoots tissue from three biological replicates were aliquoted in 0.1 mg samples. The total RNA (plant and bacterial) was extracted with the RNeasy^®^ Plant Mini Kit (Qiagen) according to the manufacturer instruction, using RLT buffer and adding a DNase incubation step (RNase-Free DNase Set, Qiagen) between the points 6 and 7 of the manufacturer’s RNA extraction protocol. The RNA samples so obtained were brought to the same concentration using Qubit BR assay (ThermoFisher Scientific), and cDNA libraries were produced using SuperScriptTM III Reverse Transcriptase Kit (Invitrogen). Each RTqPCR reaction was set up using 5 µL SensiMix SYBR Lo-ROX Kit (Meridian Bioscience^®^), 1 µL forward primer 10 µM, 1 µL reverse primer 10 µM (Primers **Supplementary Table S2**), 200 ng cDNA and sterile water to 10 µL. Each biological replicate was subdivided in three technical replicates. The PCR were performed on a LightCycler^®^ 480 System apparatus at the following conditions: 10 min at 95°C followed by 40 cycles with steps of 95°C for 15 s, 60°C for 15 s, 72°C for 15 s, and a final 2 min amplification at 72°C. Data analysis was carried out as in Banani et al., 2014. The *nifD* gene expression was investigated and assessed against the reference bacterial gene *rho* as in (Galisa et al., 2012).

### qPCR analyses

Tomato plantlets were collected from hydroponic systems and divided into 5 different anatomical zones (**Supplementary Figure S5**). The total DNA was extracted with the following protocol. 0.1 g of plant material from three biological replicates of each treatment were macerated in liquid nitrogen. Then, 600 µL of DNA extraction buffer [for 100 mL, add 20 mL of 1M TrisHCl pH 7.5, 6.25 mL of 4M NaCl, 5 mL of 0.5M EDTA, 5 mL 10% SDS] was added to each sample, incubated for 5 mins at room temperature and centrifuged at 13k x *g* for 10 minutes. 500 µL of the supernatant was mixed with 500 µL of isopropanol and incubated at room temperature for 3 mins. Samples were centrifuged at 20k x *g* for 10 mins and the supernatant was discarded. Pellets were washed once in 70% ethanol, air-dried, resuspended in 50 µL of distilled water and adjusted to the same total DNA concentration. qPCR reactions of each biological replicate were carried out in three technical replicates with the cycling protocol reported in the section above, using one primer combination from the tomato genome as reference (from the *ard2* gene) and one primer combination from the bacterial genome (from the *nifD* gene, see Primers **Supplementary Table S2**).

### Statistical analysis

All experiments were carried out at least twice (repeat numbers are stated in the figure legends) and data were analysed with Statistica 13.1 software (TIBCO Software, Palo Alto, CA, USA) and RStudio. Growth promotion in glasshouse experiments and bacterial re-isolation data were analysed using non-parametric tests. Multiple comparison analyses with either Kruskal-Wallis or through the Mann–Whitney U test followed by *post-hoc* Dunn’s test were performed to demonstrate significant differences between groups in each experiment (P ≤ 0.05). Statistical significance of RT-qPCR, qPCR analyses, and growth promotion in hydroponic systems, in which normality was met, was assessed by one-way ANOVA test followed by *post-hoc* Tukey’s HSD test at P ≤ 0.05.

### X-Gluc staining

To visualise the GUS gene expression in the pRGS561 tagged bacteria, plants were collected from the hydroponic systems at 3 to 30 dpi and incubated for 1 to 2 days in dark at 28°C in GUS staining solution [0.17 g L^-1^ (0.5 mM) Potassium Ferricyanide; 0.18 g L^-1^ (0.5 mM) Potassium Ferrocyanide; 100 mM Phosphate buffer pH 7; 1 mM EDTA pH 8; 0.8% v/v Triton X-100; 0.5 mg mL^-1^ 5-bromo-4-chloro-3-indolyl-beta-D-glucuronic acid, cyclohexyl ammonium salt (X-Gluc)]. After incubation, the staining solution was discarded and substituted with 70% ethanol and stored at room temperature until chlorophyll was completely removed from samples facilitating blue signal visualisation.

### Foliar application and protoplast isolation

Young, apical leaves of uninoculated tomato plants grown hydroponically were cut in strips approximately 5 mm wide. The strips were incubated in the dark for 5 hours at 28°C in a solution of *G. diazotrophicus* (cultured and washed as described for hydroponic inoculation) resuspended in water at 10^7^ CFUs mL^-1^. After the incubation, the leaf strips were washed twice by swirling in sterile water and conserved at 4°C with 100% humidity for microscopy analyses. Alternatively, the washed leaf strips were transferred to the digestion solution (0.1 g leaf tissue in 2 mL digestion solution) and put in gentle agitation at room temperature for one hour for protoplast isolation. The digestion solution was made with 0.3% w/v Cellulase “Onozuka R-10” (Duchefa Biochemie) and 0.4% w/v Macerozyme R-10 (Duchefa Biochemie) dissolved in Plant Protoplast Digest/Wash Solution (Sigma-Aldrich, Merck) for 10 minutes at 50°C, then supplemented with 10% v/v Viscozyme^®^ L (Sigma-Aldrich, Merck) and 1% w/v BSA. After the incubation, the protoplasts were pelleted at 500 x *g* for 4 minutes; the pellet was carefully removed from the digestion solution by pipetting and transferred to some fresh Plant Protoplast Digest/Wash Solution (Sigma-Aldrich, Merck). The protoplasts so obtained were directly observed through bright field microscopy.

### Microscopy and image analysis

Widefield fluorescence imaging of samples at the mm-scale level was carried out with a Modular Stereo Microscope for Fluorescent Imaging Leica MZ10 F coupled with Chroma’s ET GFP LP (ET480/40x, ET510 LP), allowing for excitation and detection of both GFPmut3* and DsRed-Express2 reporter proteins, or with Chroma’s ET dsRed (ET546/10x, ET595/50m). For widefield fluorescence microscopy at the µm-scale level, a Leica DM5000 B Automated Upright Microscope was employed, coupled with a L5 filter set for GFPmut3* detection (BP480/40x, BP527/30) and a N2.1 filter set for DsRed-Express2 BP detection (BP515-560, LP 590). For confocal fluorescence microscopy and z-stacks, a Leica TCS SP5 confocal microscope was employed, with a 488nm laser and “Leica/EGFP” software pre-set for imaging GFPmut3* and a 633nm laser to image plastidial chlorophyll. Sample sectioning was carried out with a 7000smz-2 Vibrotome (Campden Instruments) as in Atkinson and Wells, 2017. Z-stacks, 3D models and composite images were processed with the Fiji software using the Z Project, 3D Project and Merge Channels algorithms, respectively, leaving default settings. ET = enhanced transmission; x = excitation (filter); m = emission (filter); LP = long-pass; BP = band-pass.

## Results

### Gd WT promoted tomato growth in glasshouse experiments

The ability of the wild type strain *G. diazotrophicus* AZ0019 (Gd WT) to promote the growth of tomato plants was preliminarily tested under glasshouse conditions. *G. diazotrophicus* was applied to seeds after being resuspended in a bacterial coating adjuvant. This formulation was adopted to favour bacterial adhesion to the seeds and provide an initial carbon source for bacterial growth during the early colonisation stages. Seeds were then sown into a nutrient-rich compost and grown in a glasshouse environment. Germination rate was not affected by seed treatment with *G. diazotrophicus*, with both untreated and *G. diazotrophicus*-treated seeds showing a germination rate of around 90%.

**Figure 1:**
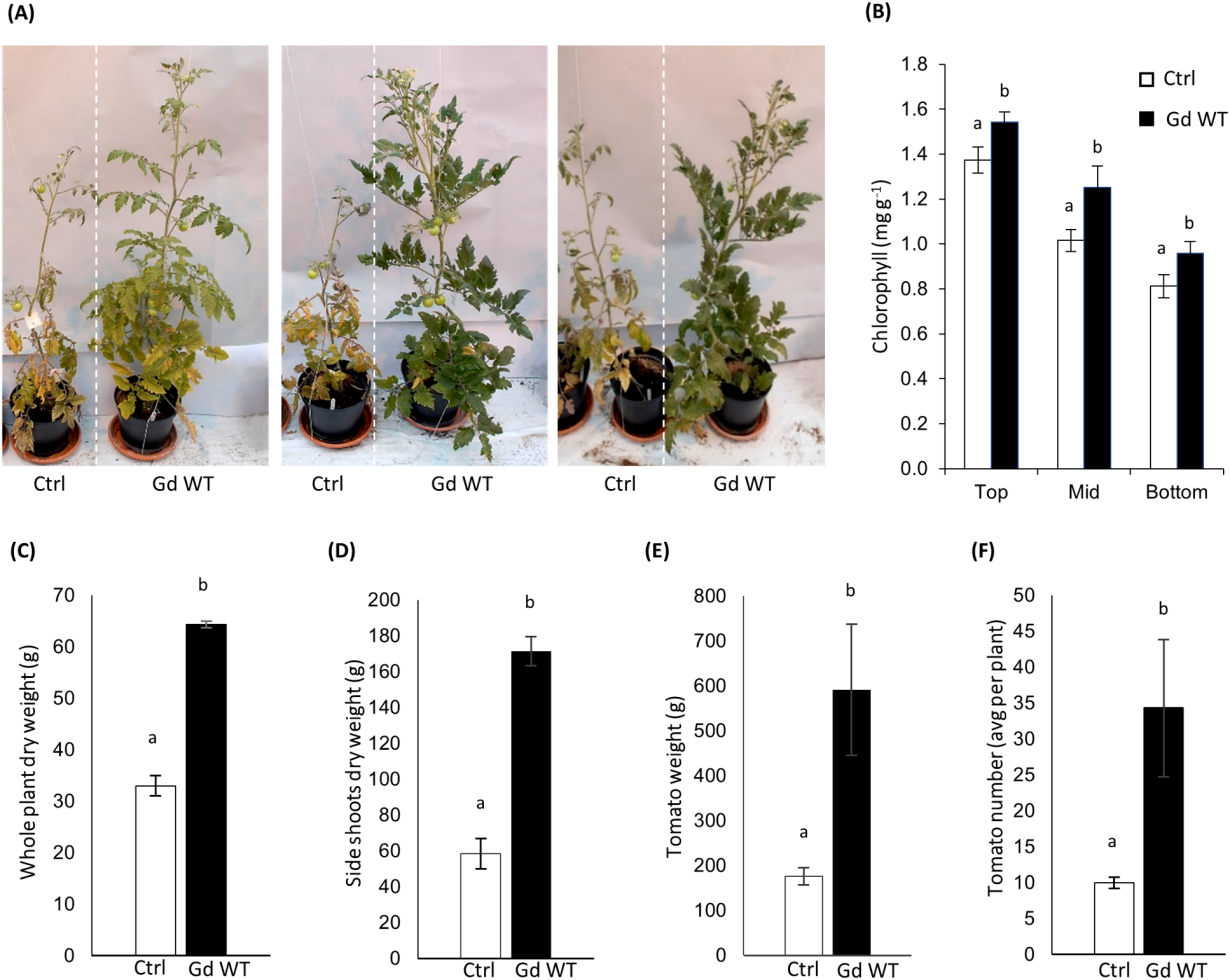
Growth promotion phenotype **(A)** four months post sowing in tomato plants inoculated with Gd WT through the seed coating technique, in comparison to mock-treated controls (Ctrl). To assess chlorophyll content **(B)**, SPAD measurements were performed on 10 different leaf blades from three parts of 4 months old tomato plants (Top, Middle, Bottom) and SPAD conversion to chlorophyll content was calculated as in Jiang et al. (2017). Dry weight (aerial parts) **(C)**, per-plant average side shoot dry biomass **(D)**, per-plant average weight **(E)** and number **(F)** of tomatoes produced are shown. N=10 for all parameters tested. The standard error of the mean is shown. Significance of differences between treatments was assessed by Kruskal-Wallis test followed by Post-Hoc Dunn’s test at P ≤ 0.05.

In sugarcane, *G. diazotrophicus*-mediated PGP comes into play as the rapid growth of crops deplete the nutrients from the soil (Boddey et al., 1991). To mimic this dynamic, the one-month-old tomato plants were re-potted in a low-nutrients substrate (Klassman Tray substrate) mixed with an equal volume of sand and routinely watered with nitrogen-free feeding. At four months post inoculation, the plant weight and fruit yield were assessed, and the side shoots dry weight and plant chlorophyll content [using a SPAD meter as in (Jiang et al., 2017)] were measured (**Figure 1**).

The in-soil experiment outcomes produced promising data (**Figure 1A**). Chlorophyll content (**Figure 1B**), whole plant dry weight (**Figure 1C**), side shoots dry weight (**Figure 1D**), and yield (**Figures 1E, F**) were significantly increased in Gd WT treated plants in comparison to mock-treated controls (Ctrl). However, the results among experimental repeats exhibited variability, even when experiments were conducted maintaining identical conditions.

An improved phenotype, although pronounced up to four months post inoculum in at least three experimental replicates and present to some extent across every further repetition, was, in most cases, visible primarily 15 days to one month after inoculation and became less pronounced during later plant growth and maturation (**Supplementary Figure S2**).

### Differential PGP of Gd *nifD^-^* compared to Gd WT in hydroponic systems

To overcome the variability of in-soil experiments and gain fine control over crucial variables in the plant-bacteria interaction, a hydroponics setup was developed for further investigations. Untreated tomato seeds cv. Moneymaker were germinated in the hydroponic system on a modified version of Hoagland solution (referred to as nutrient solution, NS) in presence of either 2 mM KNO_3_ as sole source of nitrogen (N replete condition), or no nitrogen source (zero N condition). *G. diazotrophicus* was added to the system as an inoculum in the NS at the final concentration of 10^7^ CFU mL^-1^, and incubated for three days, before being removed through NS replacement. One of the primary interests of this work was to investigate the requirement of a functional *nifD* gene in *G. diazotrophicus* for optimal plant growth promotion.

BNF in diazotrophs is catalysed by the nitrogenase complex, the catalytic core of which is encoded by the *nifHDK* polycistronic operon (Lee et al., 2000). In our study, the *nifD* gene was disrupted by partial deletion and insertion of a kanamycin resistance cassette (**Supplementary Methods S1**, **Supplementary Figure S1**), thus allowing the effect elicited by the mutant (Gd *nifD^-^*) and the Gd WT strain to be compared. *G. diazotrophicus* mutants with insertion in the *nifD* region are unable to fix nitrogen (Sevilla et al., 1998). The mutant genotype was confirmed through whole genome sequencing (**Supplementary Figure S1**) and Southern Blotting, while nitrogenase inactivation was verified through acetylene reduction assays (ARA) (**Supplementary Methods S2**, **Supplementary Figure S3**).

**Figure 2:**
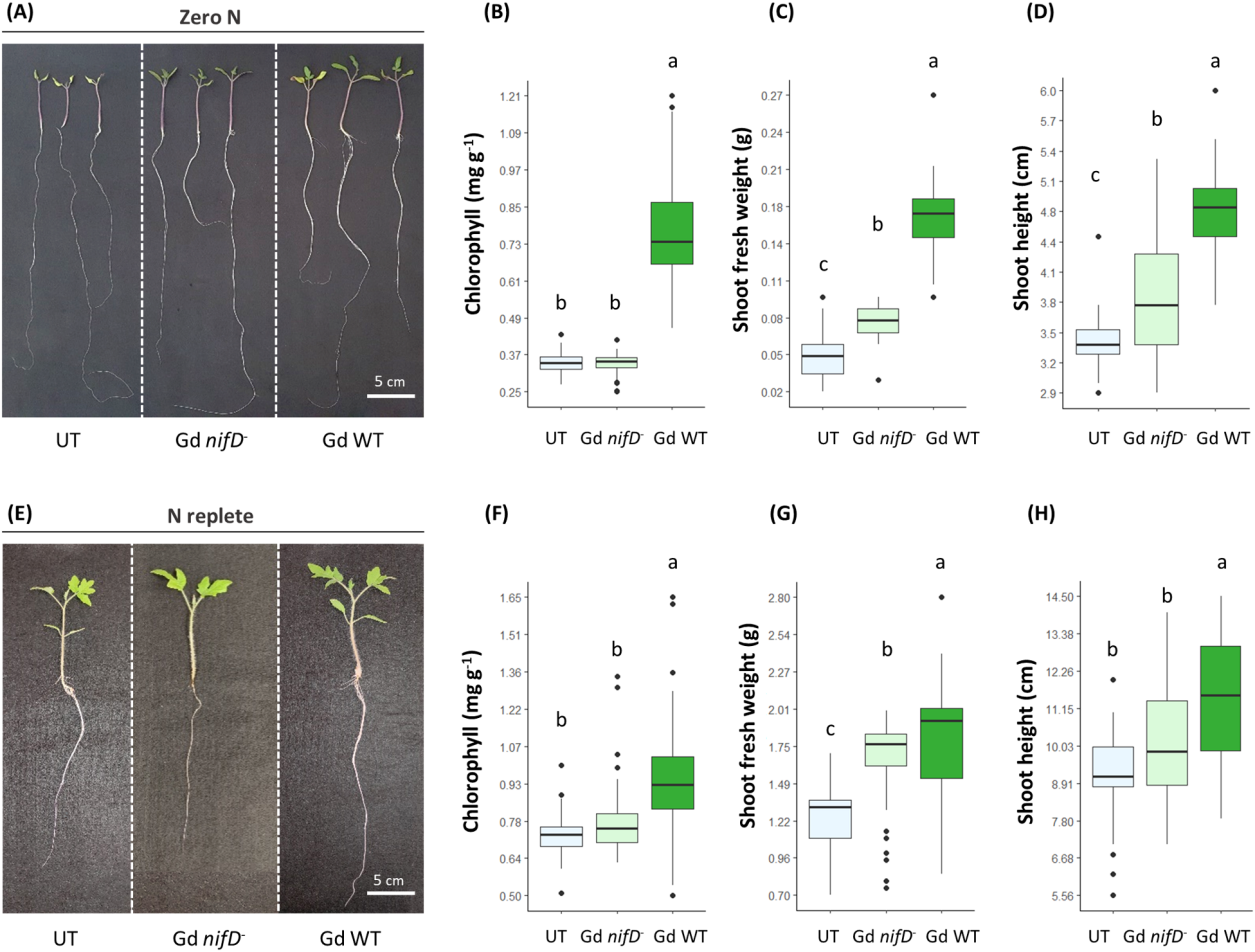
Growth promotion phenotype **(A, E)** 15 dpi in hydroponically grown tomato plants inoculated with either Gd WT or Gd *nifD^-^*, in comparison to untreated plants (UT). Chlorophyll content **(B, F)** (SPAD conversion to chlorophyll content was calculated as in Jiang et al. (2017)), shoots fresh weight **(C, G)** and shoot length **(D, H)** measurements of plants grown in either zero N **(A–D)** or N replete **(E–H)** conditions are reported. N ≥ 35 for all parameters tested. Significance of differences between treatments was assessed by one-way ANOVA test followed by Post-Hoc Tukey’s HSD test at P ≤ 0.05.

Phenotypic data from inoculated plants were collected two weeks after bacterial inoculation (15 dpi; **Figure 2**).

Regardless of the presence of nitrogen in the medium, the inoculation with either Gd *nifD^-^* or Gd WT strains led to an increase in fresh weight in comparison to uninoculated controls, with the Gd WT strain promoting an enhanced biomass accumulation compared to Gd *nifD^-^* inoculated plants (**Figures 2C, G**). Similarly, the shoot height increased when exposed to either strain under nitrogen starvation, with the wild type bacterium promoting a more pronounced effect (**Figure 2D**). However, in N replete conditions, the Gd *nifD^-^* induced shoot elongation was not significantly different to the control (**Figure 2H**). Under both nitrogen conditions, only the wild type strain promoted an increase in chlorophyll content (**Figures 2B, F**).

**Figure 3:**
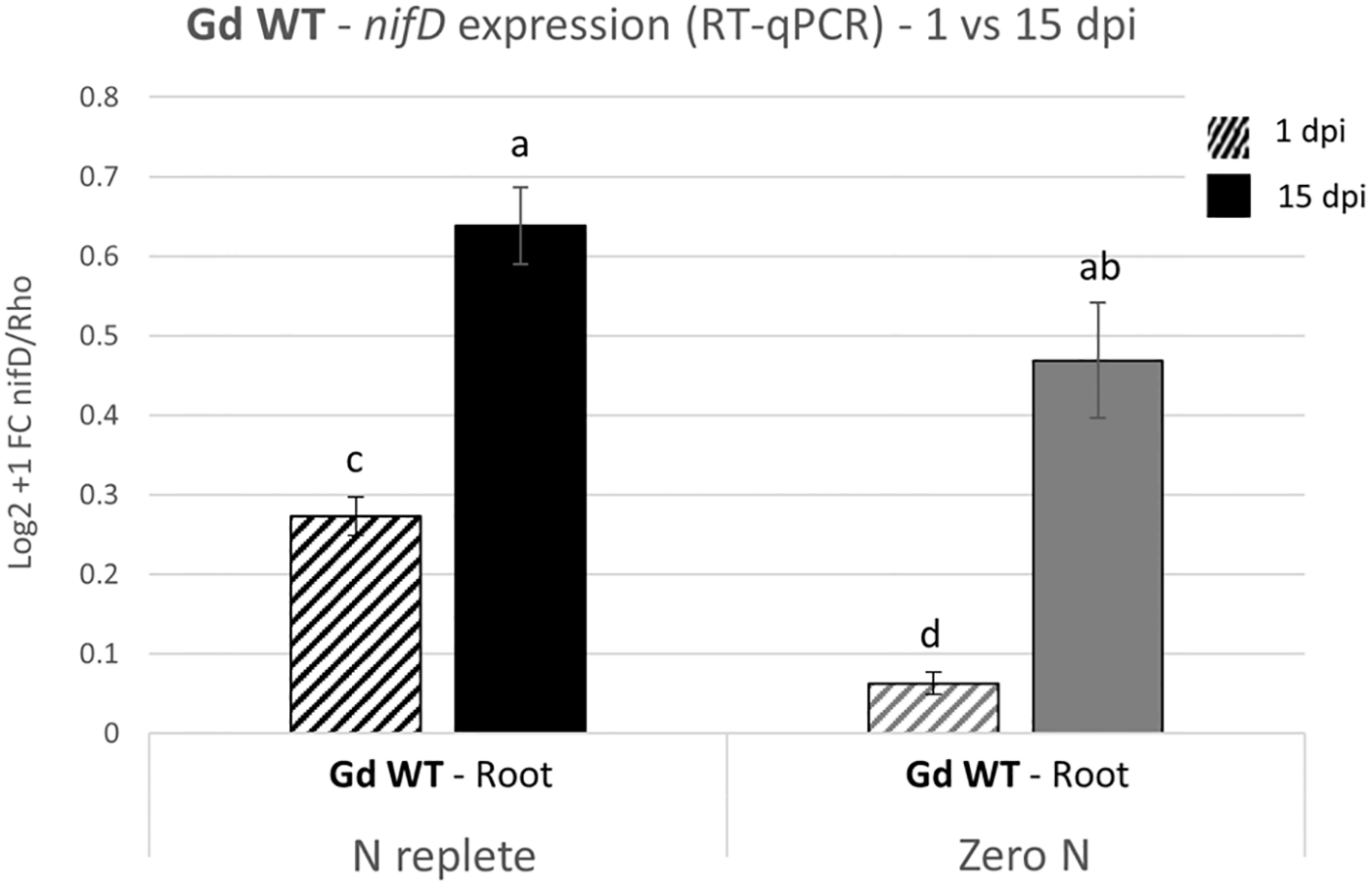
*nifD* expression analysis on hydroponically grown tomato plants inoculated with Gd WT. RT-qPCR were performed on samples collected 1 and 15 dpi (graphically represented, respectively, as stripe pattern fill and solid fill) and the expression of *nifD* was assessed as compared to the reference constitutive bacterial gene *Rho*. The Log2 + 1 of the fold change (FC) with the standard error of the mean is plotted. Significance of differences between treatments was assessed by one-way ANOVA test followed by Post-Hoc Tukey’s HSD test at P ≤ 0.05.

To confirm that transcription of the *nif* operon of Gd WT was occurring in this setup, the expression of the *nifD* gene in the root tissues of plants inoculated with Gd WT was screened through RT-qPCR at 1 and 15 dpi (**Figure 3**). The expression of *nifD* significantly increased between 1 and 15 dpi under both nitrogen conditions. Furthermore, during the early stages of interaction (1 dpi), the transcript levels were upregulated in the N replete systems compared to the zero nitrogen conditions. No signal was detected in uninoculated plants (not shown).

### Plant colonisation was comparable in *G. diazotrophicus* wild type and *nifD^-^* mutant

**Figure 4:**
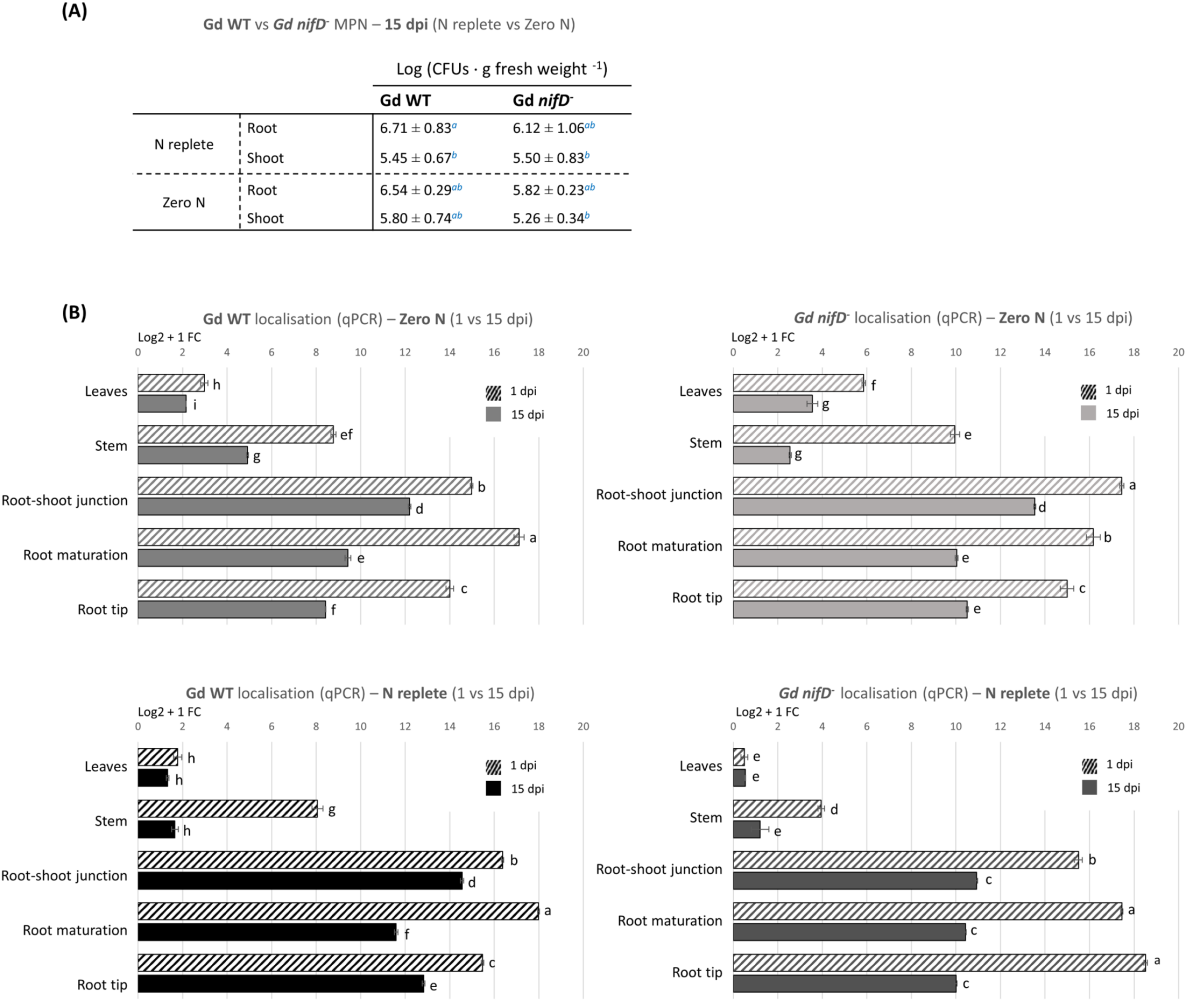
Colonisation analyses on hydroponically grown tomato plants inoculated with Gd WT or Gd *nifD* ^-^ under N replete or zero N conditions. **(A)** Reisolation of bacteria from fresh plant tissues through the Most Probable Number (MPN) method. CFUs per plant gram at 15 dpi are reported as distinguished in shoot and root. No bacteria were isolated from uninoculated controls (not shown). The standard deviation is shown. Significance of differences between treatments was assessed by Kruskal-Wallis test followed by Post-Hoc Dunn’s test at P ≤ 0.05. **(B)** qPCR analyses on plants divided into five anatomical regions (leaf, stem, root-shoot junction, maturation/differentiation zone, and root tips, **Supplementary Figure S5**). The graph represents the Log2 + 1 of the fold change (FC) of the amplified bacterial genome (from the bacterial *nifD* gene) as compared to the reference tomato genome (amplified from the tomato ard2 gene). The standard error of the mean is shown. Significance of differences between treatments was assessed by one-way ANOVA test followed by post-hoc Tukey’s HSD test at P ≤ 0.05.

To determine whether the less pronounced PGP effect induced by the mutant strain was a consequence of a poorer colonisation ability caused by the lack of a functional nitrogenase, the bacterial re-isolation rate from plant tissues was assessed for both strains. The colonisation level was evaluated through the Most Probable Number method (MPN) as CFUs of *G. diazotrophicus* per gram of fresh root and shoot (stem and leaves) material (**Figure 4A**). In this assay, bacteria that remained associated with the plant samples after washing were considered to be colonising tomato tissues, without distinguishing between epiphytic and endophytic colonisation.

No significant difference in colonisation was detected between Gd WT and Gd *nifD^-^*. Both strains displayed approximately a tenfold higher level of colonisation in the root tissues (10^6^ ± 10^1^ CFU g^-1^) in comparison to shoots (10^5^ ± 10^1^ CFU g^-1^) under N replete condition; similar colonisation rates were observed in the absence of nitrogen (**Figure 4A**). It must be emphasised that bacterial growth in hydroponics nutrient solution alone had been tested and, due to the lack of a carbon source, no growth was observed when plants were not present in the system (**Supplementary Figure S4**).

To obtain a quantification of the relative distribution of *G. diazotrophicus* throughout the plant tissues and gain insight into the potential influence of nitrogen fixation on the bacterial behaviour, DNA was extracted from the same samples and analysed through qPCR targeting the bacterial genome (**Figure 4B**).

Shoot colonisation was around one order of magnitude lower compared to the root system, confirming the trend observed through the MPN method. Furthermore, after two weeks the associated bacterial population had decreased between 15% and 75% compared to 1 dpi, especially in the stem, whereas the decline was less pronounced around roots and leaves.

At 1 dpi, roots inoculated with Gd WT were most densely colonised in the maturation/elongation zone, while the root tips showed lower colonisation than other root areas regardless of the N condition. Gd *nifD^-^* exhibited different root colonisation patterns at 1 dpi between N replete and zero N conditions; however, regardless of the nitrogen level, at 15 dpi both strains established themselves at high concentration in the root-shoot junction. Both strains, and particularly the Gd *nifD^-^* mutant, exhibited higher colonisation of the phylloplane under zero N compared to the N replete condition.

### Colonisation imaging

**Figure 5:**
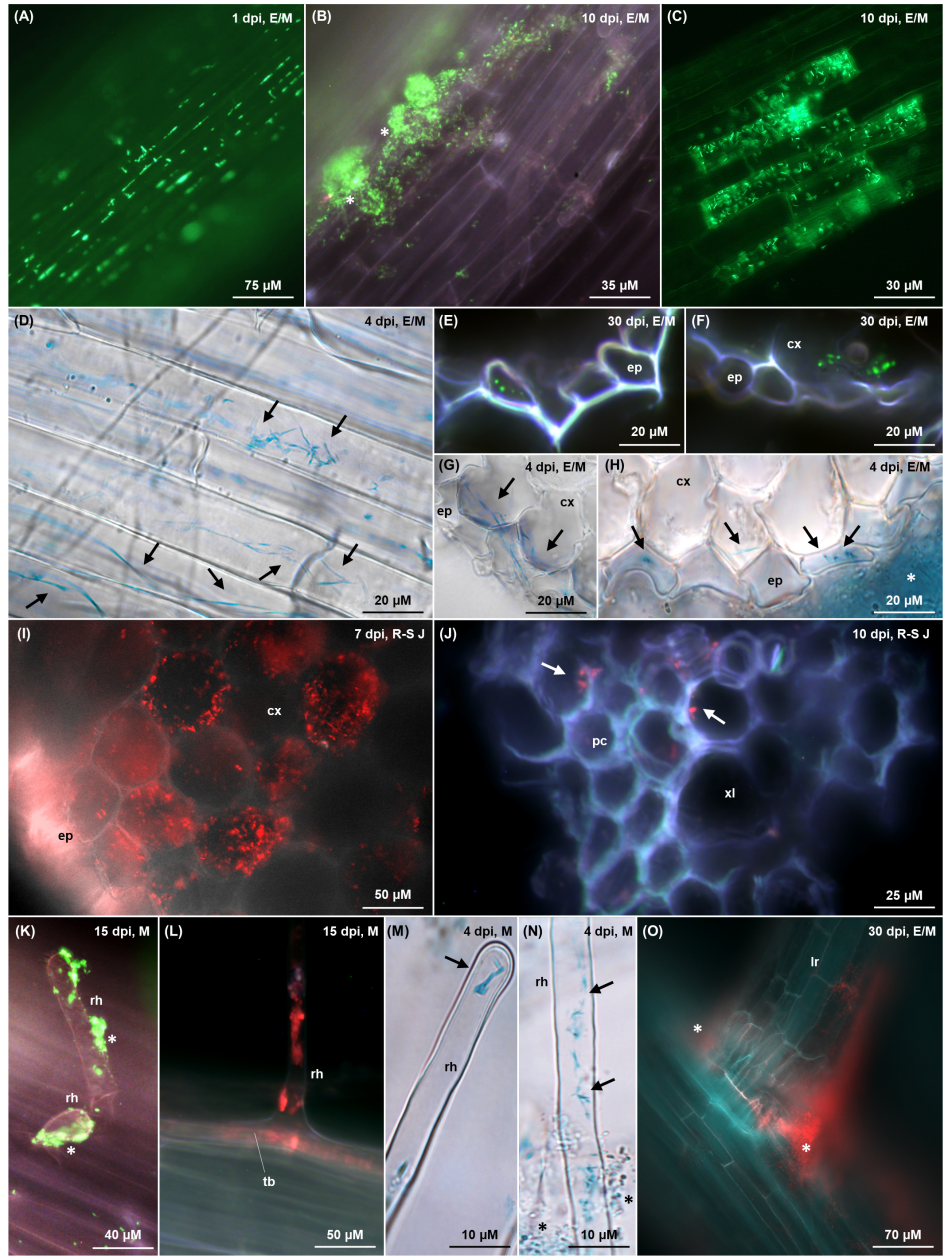
Colonisation imaging of tomato plants grown hydroponically **(A–G, I–M)** or on MS agar **(I, O)** under N replete condition, inoculated with tagged *G. diazotrophicus* strains as described in **Figures 1** and **2** respectively. Fluorescence from GFPmut3* **(A–C, E, F, K)** and dsRed-Express2 **(I, J, L, O)** is visualised as green and red signals, respectively, through *fluorescence microscopy*. Bacterial β-glucuronidase activity **(D, G, H, M, N)** is visualised as blue signals through brightfield microscopy following X-Gluc staining. In the top right corner of each picture, the timepoint of observation and anatomical zone of the root are indicated (E = elongation; M = maturation; R-S J = root-shoot junction). **(A)** Individual interspersed bacterial cells (bright green) colonising root epidermis in the elongation zone, mostly along cell wall junctions. **(B)** Bacterial biofilm (white stars) composed of rod/coccoidal cells (bright green) aggregating on root epidermis surface. **(C)** Rod-shaped bacteria colonising intracellularly a subset of epidermal cells, presumably committed to aerenchyma formation. **(D–H)** Elongated and filamentous **(G)** diazotrophicus cells (black arrows) colonising intracellularly epidermal root cells and the first cortex layer, with an epiphytic biofilm surrounding the epidermis (**H**, white star). In cross sections **(E–H)**, elongated/filamentous bacteria perpendicular to the root section appear as dots (**E**, **F**, bright green; **H**, black arrows), whereas filamentous cells traversing two adjacent cortex cells (**G**, black arrows) appear aligned parallel to the root section. **(I)** Root-shoot junction cross section showing intracellular colonisation (red) of the cortical parenchyma cells. **(J)** Rod/coccoidal bacterial cells (red, white arrows) colonising xylem vessels in the stele along the root-shoot junction. **(K–N)** Root hairs colonisation extending from the trichoblast **(L)** to the hair tip **(M)** epiphytically **(K)**, endophytically **(L, M)** and both **(N)**. Epiphytic bacteria are aggregated in biofilm (**K**, white stars; **N**, black stars) while endophytic bacteria display an elongated rod morphology (**M**, **N**, black arrows). **(O)** Crack entry from a bacterial biofilm (red) surrounding a lateral root emergence site. cx, cortex; ep, epidermis; lr, lateral root; pc, procambium; rh, root hair; tb, trichoblast; xl, xylem.

To investigate the colonisation process *in planta* in our model system, two labelled *G. diazotrophicus* strains were produced. The first strain carried a green fluorescent protein (GFP) expressing plasmid (pBBRMCS5-GFP) and a β-glucuronidase (GUS) expressing plasmid (pRGS561), with both reporter genes driven by strong constitutive promoters (see respectively Andersen et al., 1998 and Fuentes-Ramírez et al., 1999). The second tagged *G. diazotrophicus* strain carried the pICH47751 plasmid (Weber et al., 2011) harbouring a highly stable variant of the *dsRed* gene, named *dsRed-Express2* (Strack et al., 2008), under a strong constitutive P_c_ promoter from the pSW002-Pc-DsRed-Express2 vector assembled and validated by Wilton et al., 2018. The tagged strains were employed to inoculate hydroponic tomato systems under N replete conditions (as described for **Figure 2**), enabling the visualisation of plant-associated bacteria. In parallel, they were applied to excised shoot tissues from hydroponically grown tomato to investigate foliar application and enable protoplast isolation. Additionally, to complement the imaging data obtained from hydroponic cultures, colonisation was monitored in coated seeds (as described for **Figure 1**) sown in ammonium-free MS agar. In parallel to tomato, coated seeds of *A. thaliana* were grown on ammonium-free MS agar to compare the colonisation process in an alternative dicot model system (**Supplementary Figures S6**, **S7**). The tagged strains interacting with plant rhizosphere (**Figure 5**, **Supplementary Figure S6**) and phyllosphere (**Supplementary Figures S6**, **S7**) were monitored from 1 to 30 dpi.

Hydroponically grown tomatoes exhibited a colonisation pattern that was also observed in both tomato and *A. thaliana* (**Supplementary Figures S6**, **S7**) when grown on ammonium-free MS agar after seed coating. Bacteria predominantly occupied the root-shoot junction and the elongation-maturation zone, with a less dense population observed in the root tip and sporadic bacterial cells in the root cap (**Supplementary Figure S5A**), confirming the qPCR data (**Figure 4B**). When an agar substrate was present, the rhizosphere was extensively colonised by an epiphytic biofilm growing along the root-agar interface (**Supplementary Figures S6D–F**). Root epidermis was colonised by individual bacterial cells, mostly gathered along the cell wall junctions (**Figure 5A**), or clumped in large biofilms, possibly arising in nutrient rich niches (**Figures 5B, G**, white stars). Within these clusters, some filamentous cells were distinguishable among a majority of rod or coccoid-shaped bacteria (**Supplementary Figures S6D–F**, white arrows). Epidermal root cells were endophytically colonised by elongated or filamentous *G. diazotrophicus* cells, some of which stretched throughout the whole length of the host cell (**Figures 5D**, **Supplementary Figure S6C**, black arrows). Narrow biofilms were observed around lateral root emergence sites (**Figures 5O**, **Supplementary Figure S6A**, white stars), although this “crack entry” was not the main invasion strategy. Cross sections of roots showed *G. diazotrophicus* penetrating in the epidermal or exodermal layer, typically ranging between 2 to 6 of what appeared to be intracellular bacteria per epidermal cell (**Figures 5E–G**). These bacteria usually appeared as round dots, indicating the cross sectioning of elongated cells arranged parallel to the periclinal wall. Some of the filamentous cells extended from one cortex/exodermis cell to the adjacent (**Figure 5G**, black arrows), suggesting a symplastic connection. Rarely, cross section of the root-shoot junction revealed a substantial endophytic colonisation of the cortical parenchyma (**Figure 5I**). A subset of epidermal cells randomly distributed throughout the root and speculated to be degenerate aerenchyma cells, showed higher colonisation, with rod or slightly elongated bacteria co-localising with the outer periclinal cell wall and lying within the same plane, suggesting their presence between the cell wall and the plasma membrane (**Figure 5C**). Root hairs were favoured entry points throughout the whole observation period. Their colonisation initiated either at the base of the emerging hair (**Figure 5L**) upon endophytic invasion of the trichoblast, or by direct epiphytic colonisation of the hair following biofilm formation on its surface (**Figure 5K**). Most of the time, both mechanisms were observed to occur simultaneously (**Figure 5N**). Rod-shaped bacteria were observed at the tip of the hair (**Figure 5M**). Few *G. diazotrophicus* cells were found adhering on the inner cortical and vascular cell walls in the procambium and xylem vessels (**Figure 5J**).

**Figure 6:**
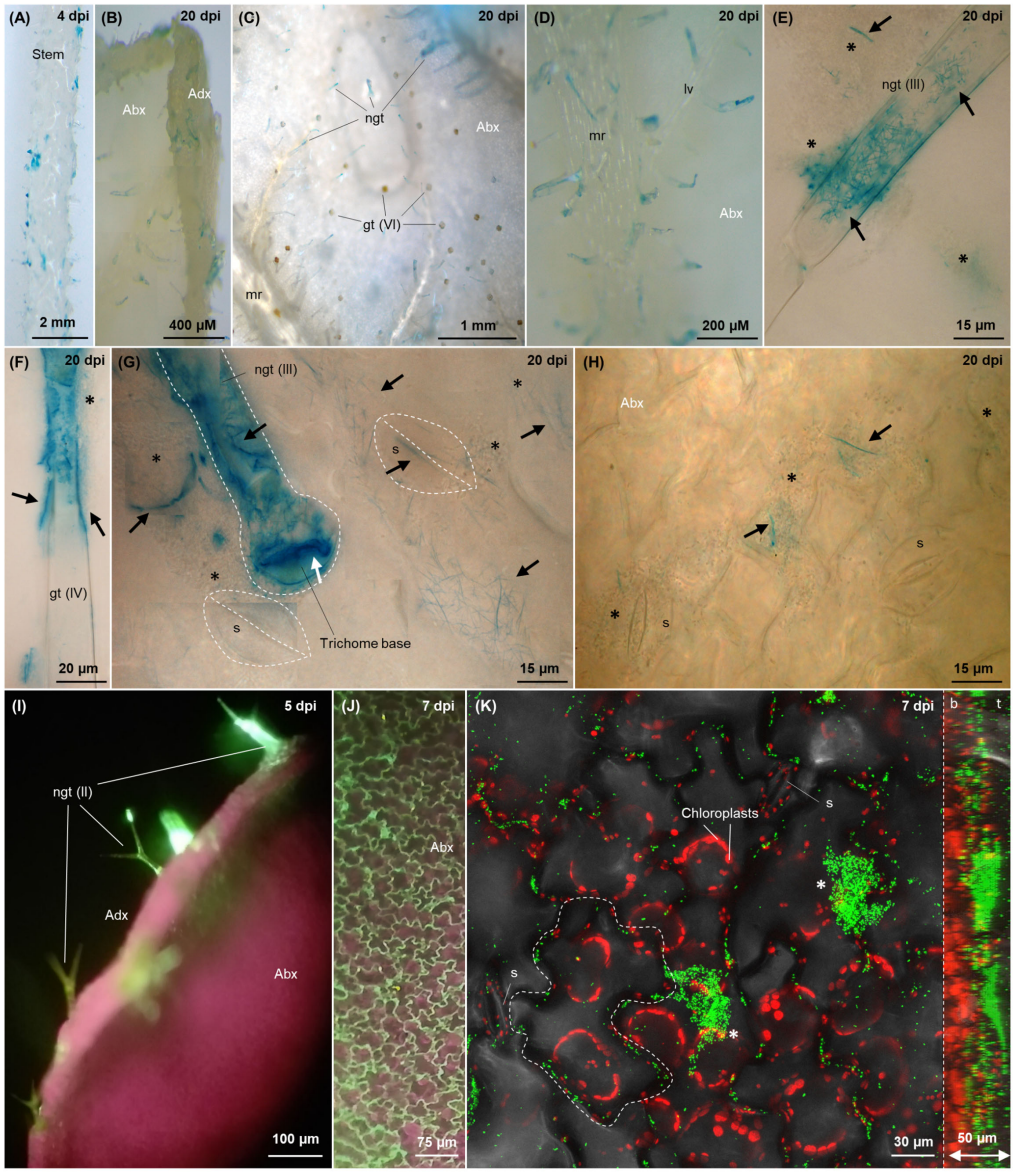
Colonisation imaging of tomato plants grown hydroponically under N replete condition, inoculated with tagged *G. diazotrophicus* strains as described in **Figure 2** **(A–H)** or treated through foliar application **(I–K)**. Fluorescence from GFPmut3* **(I–K)** is visualised as green signal, while plastidial chlorophyll is visualised in purple-red. Bacterial β-glucuronidase activity **(A–H)** is visualised as blue signals through brightfield microscopy following X-Gluc staining. In the top right corner of each picture, the timepoint of observation is indicated. **(A)** Stem colonisation at 4 dpi. **(B)** Side view of a 20-day old leaf, showing trichome colonisation (blue) on both abaxial and adaxial surfaces. **(C)** Colonisation on virtually every non-glandular trichome on the leaf abaxial surface; no blue staining is observed on glandular trichomes (orange glands). **(D)** Colonised non-glandular trichomes disseminated on the primary and secondary abaxial veins. **(E–G)** Type III non-glandular trichomes **(E, G)** and type IV glandular trichome **(F)** epi and endophytically colonised by filamentous and elongated bacterial cells, respectively (black arrows), z-stack composites. The white arrow **(G)** indicates the interface between the basal cell of the trichome stalk and the underlying epidermal cell colonised by filamentous or rod-shaped bacteria. Epiphytic biofilms of coccoidal cells surrounding the trichomes, unstained due to pRGS561 loss (but hosting a minority of stained filamentous cells, black arrows), are labelled with black stars. **(G, H)** Filamentous **(G)** diazotrophicus cells (black arrows) intracellularly colonising stomata, subsidiary cells and epidermal cells around the trichomes on the abaxial leaf surface. When not clearly visible, stomata and root hairs are demarked with white dashed line. **(I)** Side view of a leaf 5 days after foliar application, showing colonisation of Type III non-glandular trichomes (green). **(J)** Adaxial epidermis showing dense colonisation of cell wall junctions (green). **(K)** Epidermis colonisation, maximum projection of a 30 images z-stack, for a total of 50 µm depth (confocal microscopy). On the right, the lateral view of the 3D model of the z-stack is included, with its right side corresponding to the top of the z-stack (leaf surface, t) and the left side corresponding to the z-stack bottom (leaf interior, b). Chloroplasts are shown in red as a reference for evaluating the penetration of *G. diazotrophicus* into the leaf. One epidermal cell is delineated with white dashed lines to highlight the localisation of bacteria around the borders of adjacent epidermal cells. Epiphytic biofilms are labelled with white stars. Abx, abaxial leaf surface; Adx, adaxial leaf surface; ep, epidermal cell; gt, glandular trichome (the type is indicated in brackets); LV, lateral or secondary vein; MR, midrib, or primary vein; ngd, non-glandular trichome; s, stoma; sc, subsidiary cell.

In the shoot, the vast majority of non-glandular trichomes in the stem (**Figure 6A**) and leaves on both the adaxial and abaxial surface (**Figures 6B–G**) were densely colonised by elongated or filamentous *G. diazotrophicus* cells (**Figures 6E–G**), especially in correspondence with primary and secondary veins on the abaxial leaf surface (**Figure 6D**). Occasionally, colonisation was found in type I and IV glandular trichomes, which are similar in content and shape to non-glandular ones (**Figure 6F**) (McDowell et al., 2011). Trichomes were colonised on the inside by elongated bacterial cells and on the outside by filamentous cells (**Figures 6E–G**, black arrows), and were surrounded by and epiphytic biofilm of globular bacteria (**Figures 6E–G**, black stars). A high concentration of endophytic filamentous bacteria, apparently established intracellularly or in the apoplast space parallel to the periclinal wall, colonised the epidermal cells around the trichomes and stomata, including the guard and subsidiary cells (**Figure 6G**, **Supplementary Figures S7A–E**, black arrows), jointly with an epiphytic biofilm of coccoid cells interspersed with some filamentous bacteria (**Figures 6G, H**, black stars). The biofilm of coccoid cells extended in and out of stomata (**Supplementary Figures S7C, D**, white stars) and assembled along the epidermal cell wall junctions (**Supplementary Figure S7A**).

Foliar application of *G. diazotrophicus* confirmed cell wall junctions (**Figures 6J, K**) and trichomes (**Figure 6I**) to be the preferred sites for shoot colonisation by individual bacterial cells in concert with larger epiphytic aggregates (**Figure 6K**, **Supplementary Figures S7E, F** white stars).

No obvious shift in the colonisation dynamic was observed over the duration of the experiment (1 to 30 dpi) and all the different above-described mechanisms seemed to occur simultaneously at every investigated time point.

**Figure 7:**
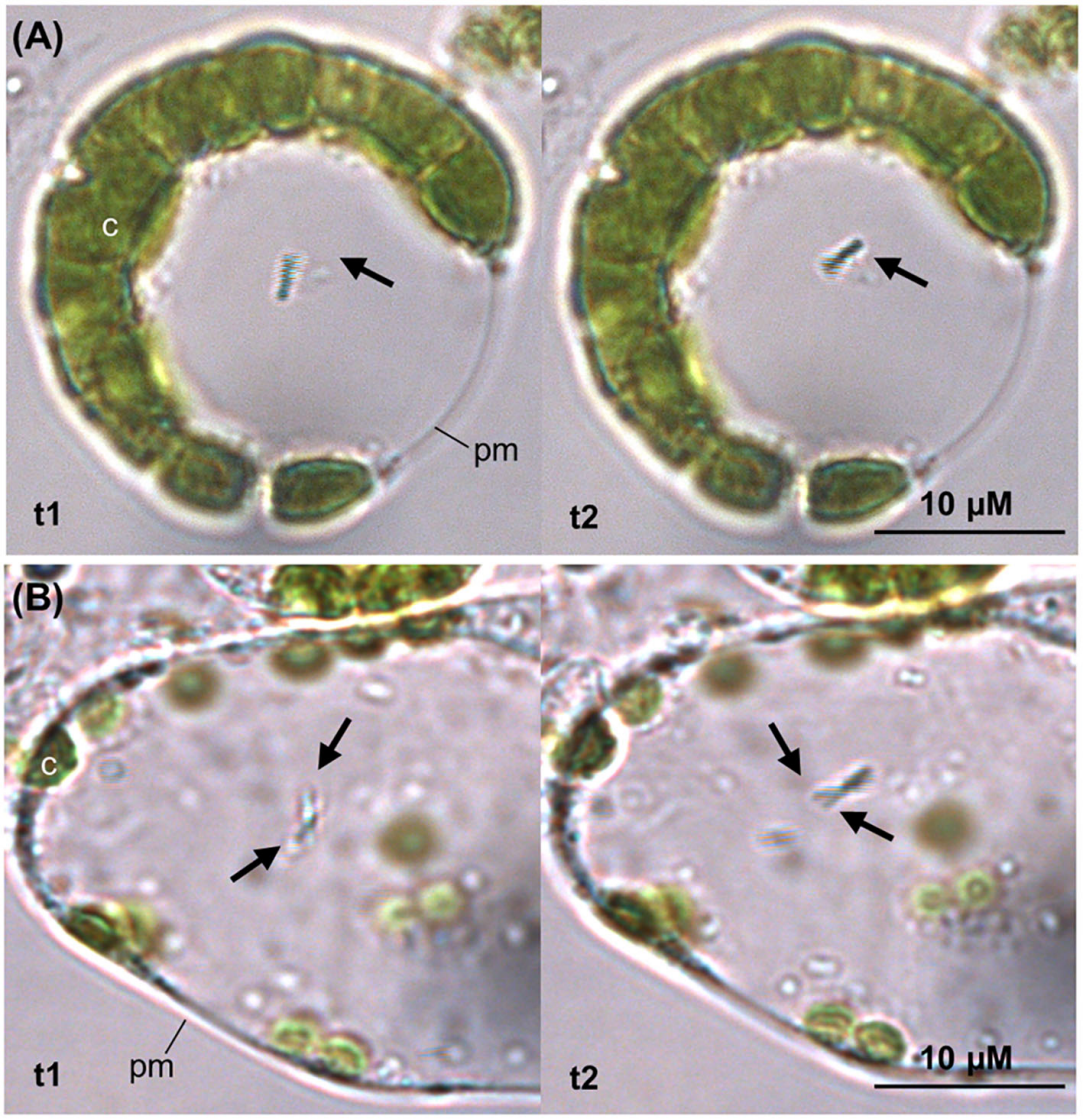
Protoplast isolation from tomato leaf strips incubated with *G. diazotrophicus*. After 5 hours of incubation of the leaf tissue with *G. diazotrophicus*, a fraction of the isolated protoplasts revealed one **(A)** to two **(B)** bacterial cells per protoplast, exhibiting swimming mobility in the cytosol. In both **(A, B)** images, the pictures on the left and on the right (t1 and t2) were taken with a 1 second interval to show the motion of the bacterium (black arrow). c, chloroplast; pm, plasma membrane.

To assess whether the putative intracellular colonisation involved bacterial uptake within the plant cytoplasm, protoplasts were isolated from tomato leaf samples treated through foliar application. A fraction of the protoplasts showed the presence of rod-shaped bacterial cells, moving inside the plasma membrane (**Figure 7**, **Supplementary Video 1**) and exhibiting the light blue coloration characteristic of *G. diazotrophicus* WT, confirming cytoplasmic uptake.

## Discussion

Plant growth promoting bacteria (PGPB) are emerging as promising tools for a new integrated and sustainable agriculture development (Brown and Saa, 2015). These microorganisms can promote plant growth through either a direct production and uptake of nutrients, or indirectly through the synthesis of molecules that influence plant development and increase its fitness and resilience against biotic and abiotic stress (Carvalho et al., 2014). *Gluconacetobacter diazotrophics* has already proved to be a good candidate as biofertiliser for its wide range of crop hosts and its ability to both fix atmospheric N_2_ and produce phytohormones such as auxins and gibberellins (Dent, 2018).

### PGP in glasshouse experiment

In this study, preliminary experiments conducted in glasshouse conditions indicated the ability of *G. diazotrophicus* AZ0019 to provide a beneficial effect on the growth of tomato plants inoculated through seed coating, sown in rich soil and repotted in low N soil to encourage nitrogen fixation. Inoculated plants showed an increased biomass, chlorophyll content and fruit yield (**Figure 1**). An improved production of side shoots and chlorophyll can be correlated to higher nitrogen availability (Puig et al., 2012; Prsa et al., 2007; Maria et al., 2017), suggesting that *G. diazotrophicus* can provide an advantage in terms of nitrogen supply.

### PGP in hydroponic system and *nifD* expression

When inoculated into crops such as tomato (Botta et al., 2013), grown in non-sterile soils under glasshouse conditions, *G. diazotrophicus* was reported to exhibit inconsistencies in the colonisation rate and PGP effect, presumably due to the inherent variability of in-soil setups. Hence, the effect elicited by *G. diazotrophicus* on tomato plants was further evaluated in a more controlled environment, i.e., in hydroponic cultures. Hydroponic based tomato production accounted for the largest global hydroponic market share of 44.2% in 2023 (Hydroponics Market Size, Share And Growth Report, 2030), making it a convenient model for both its practical and commercial interest and for the possibility of precisely controlling growth parameters. The PGP effect of the wild type bacterium (Gd WT) was compared to a BNF-impaired mutant (Gd *nifD^-^*) to assess the contribution of N nutrition to the growth stimulation. Tests were performed in nutritional stress conditions (zero N) and in presence of nitrogen in the plant nutrient solution (N replete). KNO_3_ 2 mM was chosen as sole nitrogen input in the system since this molecule is a widely employed nitrogen source for plants, which *G. diazotrophicus* is unable to utilise directly due to the lack of a nitrogen reductase (Stephan et al., 1991). Moreover, unlike other diazotrophs, the nitrogenase enzyme of *G. diazotrophicus* is not inhibited even in high nitrate concentrations (Pedraza, 2008).

We speculate that the health state of the plant can play a significant role in the establishment of a beneficial plant-bacteria association: a healthy plant would provide root exudates to boost bacterial energy metabolism, possibly fuelling BNF, since no carbon source is present in the hydroponic nutrient solution. In support of this hypothesis, the expression levels of the *nifD* gene, encoding the Mo-Fe subunit of the nitrogenase, were higher in the nitrogen-replete system compared to the nutritional stress condition at 1 dpi (**Figure 3**). This finding suggests that a minimum threshold of nitrogen, enabling a healthy plant development, can support nitrogenase transcription during early association, presumably through carbon-rich exudates available to the establishing bacterial population (Hermans et al., 2006; Sasse et al., 2018). The expression of the *nifD* gene increased over time, indicating a potential advantage for nitrogen fixation by the bacterium during the later stages of interaction. Phenotypic data from inoculated plants showed that in both zero N and N replete conditions there was a substantial increase in fresh weight and shoot height (**Figure 2**). The *nifD^-^* mutant also elicited some growth promotion, although less pronounced compared to the wild type strain (**Figure 2**), suggesting the production of plant-related hormones or other PGP activities. This observation is consistent with what was previously reported in a sugarcane model system, in which the PGP effect by the BNF mutant was restricted to N replete conditions (Sevilla et al., 1998). Notably, under both nitrogen conditions in our system, only the wild type bacterium could significantly increase chlorophyll content, suggesting a correlation between the bacterium nitrogen-fixing capabilities and nitrogen availability to the plant (Prsa et al., 2007; Maria et al., 2017). This implies that functional N-fixation is required for full PGP effects of *G. diazotrophicus*, either due to ammonium production and excretion by the bacterium for the plant uptake, or to a potential role of the fixed N in the bacterium metabolism and production of plant-related hormones (Carvalho et al., 2014). Alternatively, nitrogen fixation by the bacterium may represent an adaptation to the host plant environment, with nitrogen being released to the plant through bacterial cell lysis and mineralisation of the senescent bacterial population (White et al., 2019).

### Colonisation extent

Crucially, the different magnitude of the PGP effect exerted by the two strains was not dependent on a lower colonisation rate by the *Gd nifD^-^* strain, as both re-isolation of bacterial epiphytes and endophytes and qPCR assays found comparable bacterial abundances (**Figure 4**). Similarly, a previous study in sugarcane (Sevilla et al., 2001) reported that wild type and *nifD^-^ G. diazotrophicus* strains colonise plants to the same extent. Colonisation levels were approximately 10^6^ CFUs g^-1^ in roots and 10^5^ CFUs g^-1^ in shoots (**Figure 4A**), although the MPN method may underestimate the population of diazotrophic bacteria due to incomplete release from plant tissues and non-homogeneous suspension before plating (Silva et al., 2009). These numbers are consistent with those of a prior investigation involving inoculated tomato seedlings grown on semi-solid Fahräeus medium (Luna et al., 2012), indicating a probable natural colonisation threshold in this host crop.

qPCR analyses revealed a similar colonisation pattern between the two strains, except for minor discrepancies during early colonisation stages (**Figure 4B**). Interestingly, at the first analysed time point (i.e., one day after the substitution of the bacteria-inoculated NS with fresh sterile one) both stem and leaves were already colonised, especially under zero N condition, indicating a potential compensatory recruitment of bacteria to alleviate nutritional stress. Notably, *Gd nifD^-^* colonised leaves more efficiently than the wild type under nitrogen starvation, possibly indicating a search for nitrogen-rich niches to offset the lack of BNF (**Figure 4B**). These data suggest that transport through xylem vessels may play a central role in transferring *G. diazotrophicus* from the root system to the aerial parts of the plant, as neither apoplastic nor symplastic ascent via root cortex or epidermis appears to be sufficiently efficient to explain the presence of bacteria in leaves after only 48 hours of incubation with the inoculated NS.

The gradual decline in the bacterial population observed after two weeks (**Figure 4B**) is commonly reported for non-pathogenic endophytes, including *G. diazotrophicus*, the concentration of which was found to decline after early colonisation stages, especially under greenhouse conditions (James et al., 2001). This probably reflects the effect of dilution and spatial limitation of these microorganism ecological niches as the plants grow beyond the seedling stage (Hallmann et al., 1997). Nevertheless, *G. diazotrophicus* abundance in the phyllosphere remained relatively stable overtime, whereas after two weeks bacteria in the roots declined of around 10 to 40% compared to 1 dpi (**Figure 4B**), particularly at the root tip. The stem underwent the steepest bacterial population decrease over time, suggesting that it primarily serves as a conduit for shoot colonisation rather than a primary arrival point.

### Colonisation model

To complement qPCR data, different *G. diazotrophicus* tagged strains were used to investigate the colonisation mechanism in two monocot model systems, tomato and *A. thaliana* in fine detail. The use of three different inoculation methods (treated seeds grown on MS agar, bacterial inoculation on hydroponically grown plants and foliar application) and two nitrogen conditions (zero N and 2 mM KNO_3_) revealed the existence of common colonisation patterns.

The expression of GFP was primarily observed on external plant tissues, with occasional localisation within the first layers of the root epidermis and cortex (**Figures 5E, F**). Similarly, previous studies noted the absence of GFP fluorescence from endophytically established *G. diazotrophicus* (Sevilla and Kennedy, 2000) or *H. seropedicae* cells (Baldotto et al., 2011), possibly due to a bacterium-induced modification of the microenvironment of colonised plant cells, causing low oxygen concentration and excessively acidic pH which hinder GFP chromophore maturation (Ma et al., 2017). In contrast, due to its enhanced stability, dsRed signal was detected from bacteria colonising regions beyond the Casparian strip (**Figures 5I, J**), while GUS allowed visualisation of endophytic bacteria exhibiting a filamentous morphology (**Figure 5D, G**, **Figures 6E–H**, **Supplementary Figure 7C**). Plasmid loss or failed FP maturation were likely influenced by the combination of bacterial physiology and morphological transition, and by the inhabited endophytic microenvironment, with filamentous cells retaining the GUS plasmid but losing fluorescent ones, and coccoidal cells in biofilm exhibiting the opposite behaviour. These findings emphasise the need for complementary tagging strategies.

**Figure 8:**
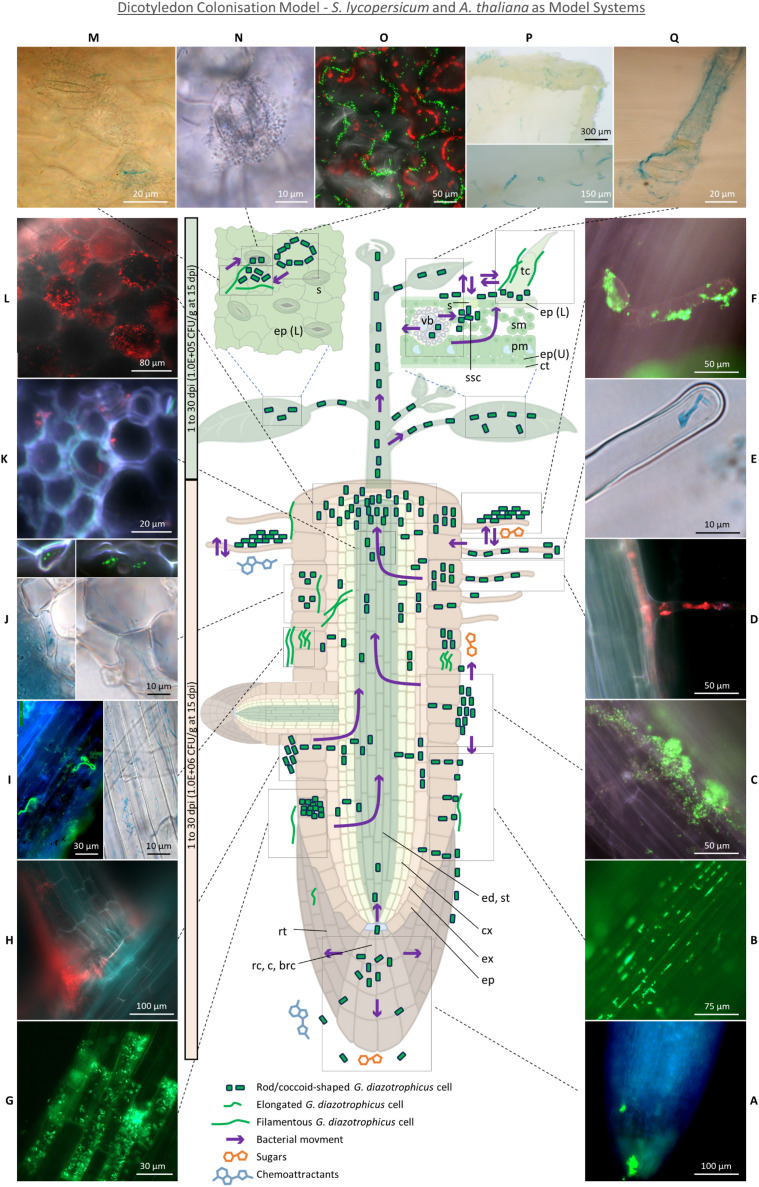
Dicotyledon Colonisation Model - *S. lycopersicum* and *A. thaliana* as model systems. **(A)** Root tip colonisation. **(B)** Epiphytic colonisation of root epidermis cell wall junctions. **(C)** Epiphytic aggregates on root epidermis. **(D)** Concomitant trichoblast and root hair colonisation. **(E)** Root hair tip intracellular colonisation. **(F)** Root hair epiphytic colonisation. **(G)** Intracellular colonisation of lysogenic root epidermal cells. **(H)** Crack entry through secondary root emergence sites. **(I)** Filamentous bacterial cell epiphytically and intracellularly colonising root epidermal cells. **(J)** Elongated or filamentous bacteria intracellularly colonising epidermis, exodermis and cortex and putatively moving across cell walls. **(K)** Rare stele colonisation by putative bacteria moving through xylem vasculature. **(L)** Dense cortex colonisation in the root-shoot junction. **(M)** Leaf epidermis colonisation by rod-shaed and filamentous bacteria around stomata. **(N)** Stomata colonisation. **(O)** Epiphytic colonisation of leaf epidermis cell wall junctions. **(P)** Abaxial and adaxial colonisation of trichomes, especially in correspondence of leaf vasculature. **(Q)** Epiphytic and endophytic non-glandular trichome colonisation. On the left, a timescale (light orange for roots, light green for shoot) indicates the timeframe in which the summarised colonisation dynamic was observed, followed by the CFUs per gram of fresh tissue reisolated through the MPN method from hydroponic cultures grown under 2 mM KNO_3_. brc, border cells; c, columella; ct, cuticle; cx, cortex; ed, endodermis; ep, epidermis (L, lower; U, upper); ex, exodermis; pm, palisade mesophyll; rc, root cap; rt, root tip; s, stomata; sm, spongy mesophyll; ssc, substomatal chamber; st, stele; tr, trichome; vb, vascular bundle.

The main root colonisation mechanisms observed in the hydroponic and MS agar setups (**Figure 8**) included epiphytic biofilms covering the root epidermis (**Figure 8C**), hairs (**Figure 8F**), and, rarely, lateral root emergence sites (**Figure 8H**), along with individual cells primarily occupying the grooves between epidermal cell wall junctions [**Figure 8B**, also previously observed in *G. diazotrophicus* inoculated rice (Sevilla and Kennedy, 2000)]. This colonisation pattern persisted throughout the experiment, suggesting that the root surface serves as a platform for bacterial multiplication and subsequent tissue invasion. Root tips (**Figure 8A**) exhibited lower colonisation levels compared to the elongation and maturation zone, despite being considered entry points for endophytic colonisation due to high exudation (Sasse et al., 2018). This may depend on tomato exudate composition *in vitro*, which includes (in descending concentrations) fructose, glucose and maltose, but in too low concentration to significantly impact the growth of rhizobacteria (Lugtenberg et al., 1999); furthermore, *G. diazotrophicus* is not able to use maltose as a C source and only exhibits moderate growth on fructose (Cavalcante and Dobereiner, 1988). Conversely, the enrichment of bacteria in the root-shoot junction (**Figure 8L**) indicates this nutrient-dense area as a primary accumulation point of bacteria during their migration toward the shoot.

In agreement with previous findings (James et al., 1994), some specific epidermal cells stood out for being heavily colonised, even amidst poorly colonised surroundings (**Figure 8G**), and, in light of their higher autofluorescence, were identified as aerenchyma tissue formed in response to the flooding stress of the hydroponic setup. Aerenchyma cells undergo the action of cell wall loosening enzymes such as cellulases, expansins and xyloglucan endo-transglycosylase (Mignolli et al., 2020), providing entry points for apoplastic colonisation and facilitating colonisation of deeper tissues beneath, as demonstrated in *H. seropedicae* B501 (Elbeltagy et al., 2001) and *R. leguminosarum* (Prayitno et al., 1999). Furthermore, the lack of a functional cytoplasm may elicit a weak defence response (James et al., 2002). This colonisation mechanism has also been reported in *H. seropedicae* Z67 (James et al., 2002), *Azoarcus* (Hurek et al., 1994) and *Serratia marcescens* (Gyaneshwar et al., 2001).

While epiphytic biofilms were mostly composed of rod or ovoidal bacteria, endophytic colonisation was mainly achieved by filamentous or elongated rod-shaped cells on epidermis (**Figures 8I**, **Supplementary Figure 6C**), exodermis (**Figure 8J**), cortex (**Figure 8L**) and root hairs (**Figures 8E, D**). Filamentation of *G. diazotrophicus* in rich media had previously been associated with high ammonium concentrations (Muthukumarasamy et al., 2002a) and was later observed in the root cortex of inoculated maize grown on MS agar (Cocking et al., 2006) and of sorghum grown on semisolid medium (Luna et al., 2010). Bacterial elongation or filamentation is a poorly understood and highly context-dependent environmental adaptation, associated to stress response, metabolic changes, surface motility, biofilm formation in hard surfaces (e.g. by the creation of a concentrated pressure force for tunnelling into semisolid environments) and spanning through redox gradients for differential respiratory rates in suboptimal oxygen conditions (Young, 2006; Karasz et al., 2022). Bacteria with filamentation capability have been associated with lignin decomposition (Karasz et al., 2022) and plant symbiosis (Finer et al., 2001). Among the signalling compounds promoting filamentation, glutamine is the strongest induction factor (Rizzo et al., 2019). Environmental and BNF-derived ammonium is assimilated by *G. diazotrophicus* into glutamine or glutamate by the glutamine synthetase and glutamine oxoglutarate aminotransferase (GOGAT) (Ureta and Nordlund, 2001), suggesting that the elongation phenotype may be induced by glutamine synthesis either upon incorporation of glutamate or ammonium from plant exudates (Paynel et al., 2001), or as a result of BNF.

Root hairs were putative entry points, found to be epiphytically and endophytically colonised at all stages of the experiment (**Figures 8D–F**). They had previously been observed to play a significant role in establishing plant-diazotrophs interaction (Mercado-Blanco and Prieto, 2012), including those of *G. diazotrophicus* and tomato (Luna et al., 2012), sorghum (Luna et al., 2010), *A. thaliana* (Rangel de Souza et al., 2016) and sugarcane (Muthukumarasamy et al., 2002b); however, their role in non-rhizobial microbe uptake remains elusive, and scarce evidence exists for their intracellular colonisation (Mercado-Blanco and Prieto, 2012). Nonetheless, they may serve as a privileged access point for endophytic invasion due to high exudation of chemoattractants (Bais et al., 2006) and to the thinner cell wall in the region of the root hair apex (**Figure 8E**) (Herburger et al., 2022). Notably, the ability of *G. diazotrophicus* to pass the plasma membrane and establish itself in the cytoplasm was suggested in this study by observing the bacterium in a protoplast system following foliar application (**Figure 7**). Tagged bacteria were found adhering, although rarely, on the inner cortical and vascular cell walls (**Figures 8K, L**), closely resembling the images produced through scanning electron microscopy by Fuentes-Ramìrez et al. in their study on sugarcane colonisation (Fuentes-Ramírez et al., 1999).This suggest that *G. diazotrophicus* might travel through the xylem sap but not accumulate in the vasculature.

After reaching the shoot, bacteria were found to be “stored” (and, possibly, subsequently secreted) within non-glandular trichomes. Symplastic transport of *G. diazotrophicus* into the trichome stalk from the underlying epidermal cell is suggested by their presence in the interface that separates these two environments (**Figure 6G**, white arrows). Lack of bacteria inside the four-disc-cells head of type VI glandular trichomes (McDowell et al., 2011) may reflect the unsuitability of this niche for bacterial invasion due to the high concentration of antimicrobial compounds such as terpenoids or myricetin (Zhang et al., 2020). Conversely, non-glandular trichomes are less metabolically active than the glandular ones; they contain photosynthates such as polysaccharide material and secondary metabolites such as phenolics (particularly polyphenols and flavonoids including naringenin, apigenin, luteolin and chrysoeriol), the concentration of which decreases as plants grow and their defence role is taken up by the developed epidermal cuticle (Karabourniotis et al., 2020). While some of these secondary metabolites such as luteolin are known to be endophyte attractants (Caetano-Anollés et al., 1988), other phenolics have antimicrobial activity, indicating the adaptation of *G. diazotrophicus* to these otherwise toxic compounds. Interestingly, trichomes contain no or very few chloroplasts (Laterre et al., 2017) and are known to develop sulphur and glutathione-dependent defence against oxidative stress (Harada et al., 2010), suggesting lower oxidation from photosynthetic reactions and, possibly offering a more favourable niche for nitrogen fixation. Moreover, tomato trichomes have been shown to import sucrose from the leaf, suggesting their colonisation to be likely the result of active chemotaxis toward both phenolic chemoattractants and carbon-rich areas. While the presence of epiphytic bacteria around the trichome bases (**Figures 6G, E**) may be the result of secretion, it is also possible that trichomes are colonised externally by bacteria emerging from adjacent stomata, which were frequently observed near colonised trichomes, following bacterial conveyance to stomatal chambers via xylem or phloem flow. It is unclear whether stomata served as the primary entry point for bacteria inhabiting epiphytically the leaf epidermis, or if they represented the outlet of a colonisation process initiated from the root system: a dynamic mechanism of ingress/egress from stomata (**Figures 8N**, **Supplementary Figures 7C, D**) and trichomes and colonisation of the shared epidermal region between these organs (**Figure 8M**) seemed to occur (as previously described for *Pseudomonas* spp (Roos and Hattingh, 1983)). Phyllospheric bacteria can exploit the highly hydrophilic environment of stomata and stomatal chambers for leaf invasion, due to the protection offered against desiccation and UV radiation (Melotto et al., 2008). Similarly, the aggregation of bacteria around epidermal cell wall junctions (**Figures 8O**, **Supplementary Figure 7A**) offers an easily accessible interface to the internal environment, as well as high exudation of sugars and aliphatic compounds and physical protection against biotic and abiotic stressors such as light and desiccation (Sivakumar et al., 2020). However, stomata have rarely been identified as colonisation locations in natural conditions (Baldotto and Olivares, 2008) or following inoculation with non-native PGPB (Compant et al., 2005). *G. diazotrophicus* seems to be one of the few diazotrophic biostimulants capable of proficient stoma colonisation, along with *Herbaspirillum seropedicae* (Baldotto et al., 2011). Reduced stomatal conductance reported in *Arabidopsis* upon *G. diazotrophicus* inoculation (Rangel de Souza et al., 2016) suggests a complex dynamic in which the chemical cross-talk between the two organisms may result in the regulation of stomata aperture to allow bacterial entrance. A similar mechanism has been described for the phytopathogen *Pseudomonas syringae*, which counteracts stomatal closure by modulating the abscisic acid-mediated mechanical regulation of guard cells (Melotto et al., 2006).

## Conclusion

Here, we have developed an experimental tomato hydroponic system and utilised it to enable a rigorous monitoring of the PGP effect and colonisation mechanism of *G. diazotrophicus* in tomato. Production of an inoculum of *G. diazotrophicus* is cheap and energetically inexpensive, and has the potential to be further scaled-up for commercial hydroponic systems. Even in the extreme zero N condition, the presence of Gd WT could alleviate the N starvation phenotype, although the bacterium-to-plant nitrogen transfer mechanism remains to be elucidated Following a starting 10^7^ CFUs mL^-1^ inoculum in the hydroponic system, 10^6^ CFUs g^-1^ of *G. diazotrophicus* were found in root tissues at 15 dpi in both N conditions, highlighting the successful establishment and active multiplication of the bacterium. The presence of 10^5^ CFUs g^-1^ of bacteria in shoots indicates a rapid migration of *G. diazotrophicus* from the root system to the plant aerial parts. Microscopy evidence indicated endophytic and possibly intracellular colonisation capabilities, with striking similarities to the colonisation strategies of *H. seropedicae* (Baldotto et al., 2011), suggesting the existence of common evolutionary adaptations that these non-nodulating diazotrophic bacteria have adopted in response to specific plant anatomical and chemical features, contributing to their success in establishing mutualistic interactions.

Further experiments will be needed to assess the fate of the bacterially fixed N inside the plant. For example, the employment of ^15^N stable isotope would allow a quantification of the bacterially fixed nitrogen and to unequivocally determine if BNF is made available to the colonised plant host. Alternatively, the identification of the *G. diazotrophicus* genes involved in auxin and gibberellin production, and a study of their relative expression in the Gd WT and *nifD^-^* mutant strain could provide insight into how N fixation is interlinked with other PGP mechanisms.

## Data Availability

The datasets presented in this study can be found in online repositories. The names of the repository/repositories and accession number(s) can be found below: https://rdmc.nottingham.ac.uk/, 10.17639/nott.7440.

## References

[B1] Andersen J. B. Sternberg C. Poulsen L. K. Bjørn S. P. Givskov M. Molin S. (1998). New unstable variants of green fluorescent protein for studies of transient gene expression in bacteria. Appl. Environ. Microbiol. 64, 2240–2246. doi: 10.1128/aem.64.6.2240-2246.1998 9603842 PMC106306

[B2] Atkinson J. A. Wells D. M. (2017). An updated protocol for high throughput plant tissue sectioning. Front. Plant Sci. 8. doi: 10.3389/fpls.2017.01721 PMC5632646 29046689

[B3] Bais H. P. Weir T. L. Perry L. G. Gilroy S. Vivanco J. M. (2006). The role of root exudates in rhizosphere interactions with plants and other organisms. Annu. Rev. Plant Biol. 57, 233–266. doi: 10.1146/annurev.arplant.57.032905.105159 16669762

[B4] Baldotto L. E. B. Olivares F. L. (2008). Phylloepiphytic interaction between bacteria and different plant species in a tropical agricultural system. Can. J. Microbiol. 54, 918–931. doi: 10.1139/W08-087 18997848

[B5] Baldotto L. E. B. Olivares F. L. Bressan-Smith R. (2011). Structural interaction between GFP-labeled diazotrophic ndophytic bacterium *Herbaspirillum seropedicae* RAM10 and pineapple lantlets ‘VitóRia.’. Braz. J. Microbiol. 42, 114. doi: 10.1590/S1517-83822011000100015 24031612 PMC3768922

[B6] Banani H. Roatti B. Ezzahi B. Giovannini O. Gessler G. Pertot I.. (2014). Characterization of resistance mechanisms activated by *Trichoderma harzianum* T39 and benzothiadiazole to downy mildew in different grapevine cultivars. Plant Pathol. 63, 334–343. doi: 10.1111/PPA.12089

[B7] Bastián F. Cohen A. Piccoli P. Luna V. Baraldi R. Bottini R. (1998). Production of indole-3-acetic acid and gibberellins A1 and A3 by *Acetobacter diazotrophicus* and *Herbaspirillum seropedicae* in chemically-defined culture media. Plant Growth Regul. 24, 7–11. doi: 10.1023/A:1005964031159/METRICS

[B8] Boddey R. M. Urquiaga S. Reis V. Döbereiner J. (1991). Biological nitrogen fixation associated with sugar cane. Plant Soil 137, 111–117. doi: 10.1007/BF02187441

[B9] Botta A. L. Santacecilia A. Ercole C. Cacchio P. Del Gallo M. (2013). *In vitro* and in *vivo* inoculation of four endophytic bacteria on *Lycopersicon esculentum* . N Biotechnol. 30, 666–674. doi: 10.1016/j.nbt.2013.01.001 23352842

[B10] Brown P. Saa S. (2015). Biostimulants in agriculture. Front. Plant Sci. 6. doi: 10.3389/fpls.2015.00671 PMC4550782 26379695

[B11] Caballero-Mellado J. Martinez-Romero E. (1994). Limited genetic diversity in the endophytic sugarcane bacterium *Acetobacter diazotrophicus* . Appl. Environ. Microbiol. 60, 1532–1537. doi: 10.1128/aem.60.5.1532-1537.1994 16349254 PMC201513

[B12] Caetano-Anollés G. Crist-Estes D. K. Bauer W. D. (1988). Chemotaxis of *Rhizobium meliloti* to the plant flavone luteolin requires functional nodulation genes. J. Bacteriol 170, 3164–3169. doi: 10.1128/JB.170.7.3164-3169.1988 3384804 PMC211264

[B13] Carvalho T. L. G. Balsemão-Pires E. Saraiva R. M. Ferreira P. C. G. Hemerly A. S. (2014). Nitrogen signalling in plant interactions with associative and endophytic diazotrophic bacteria. J. Exp. Bot. 65, 5631–5642. doi: 10.1093/jxb/eru319 25114015

[B14] Cavalcante V. A. Dobereiner J. (1988). A new acid-tolerant nitrogen-fixing bacterium associated with sugarcane. Plant Soil 108, 23–31. doi: 10.1007/BF02370096

[B15] Coale T. H. Loconte V. Turk-Kubo K. A. Vanslembrouck B. Kwan W. Mak E.. (2024). Nitrogen-fixing organelle in a marine alga. Science 384, 217–222. doi: 10.1126/science.adk1075 38603509

[B16] Cochran W. G. (1950). Estimation of bacterial densities by means of the “Most probable number. Biometrics 6, 105–116. doi: 10.2307/3001491 15420239

[B17] Cocking E. C. Stone P. J. Davey M. R. (2006). Intracellular colonization of roots of Arabidopsis and crop plants by *Gluconacetobacter diazotrophicus* . In Vitro Cell. Dev. Biol. - Plant 42, 74–82. doi: 10.1079/IVP2005716

[B18] Compant S. Reiter B. Sessitsch A. Nowak J. Clément C. Barka E. A. (2005). Endophytic colonization of *Vitis vinifera L.* by plant growth-promoting bacterium Burkholderia sp. train PsJN. Appl. Environ. Microbiol. 71, 1685. doi: 10.1128/AEM.71.4.1685-1693.2005 15811990 PMC1082517

[B19] Demarre G. Guérout A. M. Matsumoto-Mashimo C. Rowe-Magnus D. A. Marlière P. Mazel D. (2005). A new family of mobilizable suicide plasmids based on broad host range R388 plasmid (IncW) and RP4 plasmid (IncPα) conjugative machineries and their cognate *Escherichia coli* host strains. Res. Microbiol. 156, 245–255. doi: 10.1016/j.resmic.2004.09.007 15748991

[B20] Dent D. (2018). “Non-nodular endophytic bacterial symbiosis and the nitrogen fixation of *gluconacetobacter diazotrophicus* ,” in Symbiosis (London, United Kingdom: InTech). doi: 10.5772/intechopen.75813

[B21] Dong Z. McCully M. E. Canny M. J. (1997). Does *acetobacter diazotrophicus* live and move in the xylem of sugarcane stems? Anatomical and physiological data. Ann. Bot. 80, 147–158. doi: 10.1006/anbo.1997.0426

[B22] Elbeltagy A. Nishioka K. Sato T. Suzuki H. Ye B. Hamada T.. (2001). Endophytic colonization and in planta nitrogen fixation by a Herbaspirillum sp. isolated from wild rice species. Appl. Environ. Microbiol. 67, 5285–5293. doi: 10.1128/AEM.67.11.5285-5293.2001 11679357 PMC93302

[B23] Erisman J. Sutton M. Galloway J. Klimont Z. Winiwarter W. (2008). How a century of ammonia synthesis changed the world. Nat. Geosci. 1, 636–639. doi: 10.1038/ngeo325

[B24] FAOSTAT. Available online at: https://www.fao.org/faostat/en/compare (Accessed 5.6.24).

[B25] Finer K. R. Larkin K. M. Martin B. J. Finer J. J. (2001). Proximity of Agrobacterium to living plant tissues induces conversion to a filamentous bacterial form. Plant Cell Rep. 20, 250–255. doi: 10.1007/S002990100315/METRICS

[B26] Fuentes-Ramírez L. E. Caballero-Mellado J. Sepúlveda J. Martínez-Romero E. (1999). Colonization of sugarcane by *Acetobacter diazotrophicus* is inhibited by high N-fertilization. FEMS Microbiol. Ecol. 29, 117–128. doi: 10.1111/j.1574-6941.1999.tb00603.x

[B27] Fuentes-Ramirez L. E. Jimenez-Salgado T. Abarca-Ocampo I. R. Caballero-Mellado J. (1993). *Acetobacter diazotrophicus*, an indoleacetic acid producing bacterium isolated from sugarcane cultivars of México. Plant Soil 154, 145–150. doi: 10.1007/BF00012519

[B28] Galisa P. S. da Silva H. A. P. Macedo A. V. M. Reis V. M. Vidal M. S. Baldani J. I.. (2012). Identification and validation of reference genes to study the gene expression in *Gluconacetobacter diazotrophicus* grown in different carbon sources using RT-qPCR. J. Microbiol. Methods 91, 1–7. doi: 10.1016/j.mimet.2012.07.005 22814372

[B29] Gyaneshwar P. James E. K. Mathan N. Reddy P. M. Reinhold-Hurek B. Ladha J. K. (2001). Endophytic colonization of rice by a diazotrophic strain of *Serratia marcescens* . J. Bacteriol 183, 2634–2645. doi: 10.1128/JB.183.8.2634-2645.2001 11274124 PMC95181

[B30] Hallmann J. Quadt-Hallmann A. Mahaffee W. F. Kloepper J. W. (1997). Bacterial endophytes in agricultural crops. Can. J. Microbiol. 43, 895–914. doi: 10.1139/m97-131

[B31] Harada E. Kim J. A. Meyer A. J. Hell R. Clemens S. Choi Y. E. (2010). Expression profiling of tobacco leaf trichomes identifies genes for biotic and abiotic stresses. Plant Cell Physiol. 51, 1627–1637. doi: 10.1093/PCP/PCQ118 20693332

[B32] Herburger K. Schoenaers S. Vissenberg K. Mravec J. (2022). Shank-localized cell wall growth contributes to Arabidopsis root hair elongation. Nat. Plants 8, 1222–1232. doi: 10.1038/S41477-022-01259-Y 36303011

[B33] Hermans C. Hammond J. P. White P. J. Verbruggen N. (2006). How do plants respond to nutrient shortage by biomass allocation? Trends Plant Sci. 11, 610–617. doi: 10.1016/j.tplants.2006.10.007 17092760

[B34] Hurek T. Reinhold-Hurek B. Van Montagu M. Kellenberger E. (1994). Root colonization and systemic spreading of Azoarcus sp. strain BH72 in grasses. J. Bacteriol 176, 1913–1923. doi: 10.1128/jb.176.7.1913-1923.1994 8144457 PMC205294

[B35] Hydroponics Market Size, Share And Growth Report (2030). Available online at: https://www.grandviewresearch.com/industry-analysis/hydroponics-market (Accessed 5.6.24).

[B36] Imran A. Hakim S. Tariq M. Nawaz M. S. Laraib I. Gulzar U.. (2021). Diazotrophs for lowering nitrogen pollution crises: looking deep into the roots. Front. Microbiol. 12. doi: 10.3389/fmicb.2021.637815 PMC8180554 34108945

[B37] James E. K. Gyaneshwar P. Mathan N. Barraquio W. L. Reddy P. M. Iannetta P. P. M.. (2002). Infection and colonization of rice seedlings by the plant growth-promoting bacterium *herbaspirillum seropedicae* Z67. Mol. Plant Microbe Interact. 15, 894–906. doi: 10.1094/MPMI.2002.15.9.894 12236596

[B38] James E. K. Olivares F. L. de Oliveira A. L. M. dos Reis F. B. Jr. da Silva L. G. Reis V. M. (2001). Further observations on the interaction between sugar cane and *Gluconacetobacter diazotrophicus* under laboratory and greenhouse conditions1. J. Exp. Bot. 52, 747–760. doi: 10.1093/jexbot/52.357.747 11413211

[B39] James E. K. Reis V. M. Olivares F. L. Baldani J. I. Döbereiner J. (1994). Infection of sugar cane by the nitrogen-fixing bacterium *Acetobacter diazotrophicus* . J. Exp. Bot. 45, 757–766. doi: 10.1093/jxb/45.6.757

[B40] Jensen M. H. Malter A. (1995). Protected Agriculture: A Global Review (Washington, DC: World Bank Technical Paper), 253.

[B41] Jiang C. Johkan M. Hohjo M. Tsukagoshi S. Maruo T. (2017). A correlation analysis on chlorophyll content and SPAD value in tomato leaves. HortResearch 71, 37–42. doi: 10.20776/S18808824-71-P37

[B42] Jimenez-Salgado T. Fuentes-Ramirez L. E. Tapia-Hernandez A. Mascarua-Esparza M. A. Martinez-Romero E. Caballero-Mellado J. (1997). *Coffea arabica L.*, a new host plant for *Acetobacter diazotrophicus*, and isolation of other nitrogen-fixing acetobacteria. Appl. Environ. Microbiol. 63, 3676–3683. doi: 10.1128/aem.63.9.3676-3683.1997 9293018 PMC168673

[B43] Karabourniotis G. Liakopoulos G. Nikolopoulos D. Bresta P. (2020). Protective and defensive roles of non-glandular trichomes against multiple stresses: structure–function coordination. J. For Res. (Harbin) 31, 1–12. doi: 10.1007/S11676-019-01034-4/FIGURES/2

[B44] Karasz D. C. Weaver A. I. Buckley D. H. Wilhelm R. C. (2022). Conditional filamentation as an adaptive trait of bacteria and its ecological significance in soils. Environ. Microbiol. 24, 1–17. doi: 10.1111/1462-2920.15871 34929753

[B45] Laterre R. Pottier M. Remacle C. Boutry M. (2017). Photosynthetic trichomes contain a specific rubisco with a modified pH-dependent activity. Plant Physiol. 173, 2110. doi: 10.1104/PP.17.00062 28250069 PMC5373067

[B46] Lee S. Reth A. Meletzus D. Sevilla M. Kennedy C. (2000). Characterization of a major cluster of nif, fix, and associated genes in a sugarcane endophyte, *Acetobacter diazotrophicus* . J. Bacteriol 182, 7088–7091. doi: 10.1128/JB.182.24.7088-7091.2000 11092875 PMC94840

[B47] Lugtenberg B. J. Kravchenko L. V. Simons M. (1999). Tomato seed and root exudate sugars: composition, utilization by Pseudomonas biocontrol strains and role in rhizosphere colonization. Environ. Microbiol. 1, 439–446. doi: 10.1046/J.1462-2920.1999.00054.X 11207764

[B48] Luna M. F. Aprea J. Crespo J. M. Boiardi J. L. (2012). Colonization and yield promotion of tomato by *Gluconacetobacter diazotrophicus* . Appl. Soil Ecol. 61, 225–229. doi: 10.1016/j.apsoil.2011.09.002

[B49] Luna M. F. Galar M. L. Aprea J. Molinari M. L. Boiardi J. L. (2010). Colonization of sorghum and wheat by seed inoculation with *Gluconacetobacter diazotrophicus* . Biotechnol. Lett. 32, 1071–1076. doi: 10.1007/s10529-010-0256-2 20361236

[B50] Ma Y. Sun Q. Smith S. C. (2017). The mechanism of oxidation in chromophore maturation of wild-type green fluorescent protein: a theoretical study. Phys. Chem. Chem. Phys. 19, 12942–12952. doi: 10.1039/C6CP07983K 28480935

[B51] Manchanda M. Garg N. (2007). Endomycorrhizal and rhizobial symbiosis: How much do they share?. J. Plant Interact. 2, 79–88. doi: 10.1080/17429140701558000

[B52] Maria O. Sueli M. Hiago F. Lisboa C. (2017). Relative chlorophyll index on doses of nitrogen fertilization for cherry tomato culture. Afr J. Agric. Res. 12, 2946–2953. doi: 10.5897/AJAR2016.12051

[B53] McDowell E. T. Kapteyn J. Schmidt A. Li C. Kang J. H. Descour A.. (2011). Comparative functional genomic analysis of solanum glandular trichome types. Plant Physiol. 155, 524–539. doi: 10.1104/PP.110.167114 21098679 PMC3075747

[B54] Melotto M. Underwood W. Koczan J. Nomura K. He S. Y. (2006). Plant stomata function in innate immunity against bacterial invasion. Cell 126, 969–980. doi: 10.1016/J.CELL.2006.06.054 16959575

[B55] Melotto M. Underwood W. Sheng Y. H. (2008). Role of stomata in plant innate immunity and foliar bacterial diseases. Annu. Rev. Phytopathol. 46, 101–122. doi: 10.1146/ANNUREV.PHYTO.121107.104959 18422426 PMC2613263

[B56] Mercado-Blanco J. Prieto P. (2012). Bacterial endophytes and root hairs. Plant Soil 361, 301–306. doi: 10.1007/S11104-012-1212-9/FIGURES/1

[B57] Mignolli F. Todaro J. S. Vidoz M. L. (2020). Internal aeration and respiration of submerged tomato hypocotyls are enhanced by ethylene-mediated aerenchyma formation and hypertrophy. Physiol. Plant 169, 49–63. doi: 10.1111/PPL.13044 31688957

[B58] Muthukumarasamy R. Revathi G. Loganathan P. (2002a). Effect of inorganic N on the population, in *vitro* colonization and morphology of *Acetobacter diazotrophicus* (syn. *Gluconacetobacter diazotrophicus*). Plant Soil 243, 91–102. doi: 10.1023/A:1019963928947

[B59] Muthukumarasamy R. Revathi G. Seshadri S. Lakshminarasimhan C. (2002b). *Gluconacetobacter diazotrophicus* (syn. *Acetobacter diazotrophicus*), a promising diazotrophic endophyte in tropics. Curr. Sci. 83, 137–145.

[B60] Paula M. A. Reis V. M. Dobereiner J. (1991). Interactions of *Glomus clarum* with *Acetobacter diazotrophicus* in infection of sweet potato (*Ipomoea batatas*), sugarcane (*Saccharum* spp.) and sweet sorghum (*Sorghum vulgare*). Biol. Fertil Soils 11, 111–115. doi: 10.1007/bf00336374

[B61] Paynel F. Murray P. J. Bernard Cliquet J. (2001). Root exudates: A pathway for short-term N transfer from clover and ryegrass. Plant Soil 229, 235–243. doi: 10.1023/A:1004877214831/METRICS

[B62] Pedraza R. O. (2008). Recent advances in nitrogen-fixing acetic acid bacteria. Int. J. Food Microbiol. 125, 25–35. doi: 10.1016/j.ijfoodmicro.2007.11.079 18177965

[B63] Prayitno J. Stefaniak J. McIver J. Weinman J. J. Dazzo F. B. Ladha J. K.. (1999). Interactions of rice seedlings with bacteria isolated from rice roots. Aust. J. Plant Physiol. 26, 521–535. doi: 10.1071/PP98090

[B64] Prsa I. Stampar F. Vodnik D. Veberic R. (2007). Influence of nitrogen on leaf chlorophyll content and photosynthesis of ‘Golden Delicious’ apple. Acta Agric. Scand. B Soil Plant Sci. 57, 283–289. doi: 10.1080/09064710600982878

[B65] Puig J. Pauluzzi G. Guiderdoni E. Gantet P. (2012). Regulation of shoot and root development through mutual signaling. Mol. Plant 5, 974–983. doi: 10.1093/mp/sss047 22628542

[B66] Rangel de Souza A. L. S. De Souza S. A. De Oliveira M. V. V. Ferraz T. M. Figueiredo F.A.M.M.A. Da Silva N. D.. (2016). Endophytic colonization of *Arabidopsis thaliana* by *Gluconacetobacter diazotrophicus* and its effect on plant growth promotion, plant physiology, and activation of plant defense. Plant Soil 399, 257–270. doi: 10.1007/s11104-015-2672-5

[B67] Reed S. C. Cleveland C. C. Townsend A. R. (2011). Functional ecology of free-living nitrogen fixation: A contemporary perspective. Annu. Rev. Ecology Evolution Systematics 42, 489–512. doi: 10.1146/annurev-ecolsys-102710-145034

[B68] Restrepo G. Sánchez O. Marulanda Moreno S. Galeano N. Taborda G. (2017). African Journal of Biotechnology Evaluation of plant-growth promoting properties of *Gluconacetobacter diazotrophicus* and *Gluconacetobacter sacchari* isolated from sugarcane and tomato in West Central region of Colombia. Afr J. Biotechnol. 16, 1619–1629. doi: 10.5897/AJB2017.16016

[B69] Rizzo M. G. Nicolò M. S. Franco D. De Plano L. M. Chines V. Moscato F.. (2019). Glutamine-induced filamentous cells of *Pseudomonas mediterranea* CFBP-5447T as producers of PHAs. Appl. Microbiol. Biotechnol. 103, 9057–9066. doi: 10.1007/S00253-019-10144-2 31659417

[B70] Roos I. M. M. Hattingh M. J. (1983). Scanning Electron Microscopy of *Pseudomonas syringae* pv, morsprunorum on Sweet Cherry Leaves. J. Phytopathol. 108, 18–25. doi: 10.1111/J.1439-0434.1983.TB00559.X

[B71] Santi C. Bogusz D. Franche C. (2013). Biological nitrogen fixation in non-legume plants. Ann. Bot. 111, 743–767. doi: 10.1093/aob/mct048 23478942 PMC3631332

[B72] Sasse J. Martinoia E. Northen T. (2018). Feed your friends: do plant exudates shape the root microbiome? Trends Plant Sci. 23, 25–41. doi: 10.1016/J.TPLANTS.2017.09.003 29050989

[B73] Sevilla M. Burris R. H. Gunapala N. Kennedy C. (2001). Comparison of benefit to sugarcane plant growth and 15N2 incorporation following inoculation of sterile plants with *Acetobacter diazotrophicus* wild-type and Nif- mutant strains. Mol. Plant-Microbe Interact. 14, 358–366. doi: 10.1094/MPMI.2001.14.3.358 11277433

[B74] Sevilla M. De Oliveira A. Baldani I. Kennedy C. (1998). Contributions of the bacterial endophyte *Acetobacter diazotrophicus* to sugarcane nutrition: A preliminary study. Symbiosis 25, 181–191.

[B75] Sevilla M. Kennedy (2000). Colonization of rice and other cereals by Acetobacter diazotrophicus an endophyte of sugarcane from The quest for nitrogen fixation in rice. Eds. Ladha J. K. Reddy P. M. (Los Baños, Philippines: International Rice Research Institute), 151–165.

[B76] Silva L. Boddey R. Reis V. (2009). Quantification of natural populations of *Gluconacetobacter diazotrophicus* and Herbaspirillum spp. In sugar cane (*Saccharum* spp.) Using different polyclonal antibodies. Braz. J. Microbiol. 40, 866–878. doi: 10.1590/S1517-838220090004000018 24031435 PMC3768561

[B77] Sivakumar N. Sathishkumar R. Selvakumar G. Shyamkumar R. Arjunekumar K. (2020). Phyllospheric microbiomes: diversity, ecological significance, and biotechnological applications. Plant Microbiomes Sustain. Agric. 25, 113. doi: 10.1007/978-3-030-38453-1_5

[B78] Stephan M. P. Oliveira M. Teixeira K. R. S. Martinez-Drets G. Döbereiner J. (1991). Physiology and dinitrogen fixation of *Acetobacter diazotrophicus* . FEMS Microbiol. Lett. 77, 67–72. doi: 10.1111/j.1574-6968.1991.tb04323.x

[B79] Strack R. L. Strongin D. E. Bhattacharyya D. Tao W. Berman A. Broxmeyer H. E.. (2008). A noncytotoxic DsRed variant for whole-cell labeling. Nat. Methods 5, 955–957. doi: 10.1038/nmeth.1264 18953349 PMC4107390

[B80] Tapia-Hernández A. Bustillos-Cristales M. R. Jiménez-Salgado T. Caballero-Mellado J. Fuentes-Ramírez L. E. (2000). Natural Endophytic Occurrence of A*cetobacter diazotrophicus* in pineapple plants. Microb. Ecol. 39, 49–55. doi: 10.1007/s002489900190 10790517

[B81] Tian G. Pauls P. Dong Z. Reid L. M. Tian L. (2009). Colonization of the nitrogen-fixing bacterium *Gluconacetobacter diazotrophicus* in a large number of Canadian corn plants. Can. J. Plant Sci. 89, 1009–1016. doi: 10.4141/CJPS08040

[B82] Ureta A. Nordlund S. (2001). Glutamine synthetase from *Acetobacter diazotrophicus*: properties and regulation. FEMS Microbiol. Lett. 202, 177–180. doi: 10.1111/j.1574-6968.2001.tb10800.x 11520611

[B83] Vitousek P. M. Aber A. J. Howarth R. W. Likens G. E. Matson P. A. Schindler D. Q. (2011). Human alteration of the global nitrogen cycle: sources and consequences. Ecol. Appl. 7, 737–750. doi: 10.1890/1051-0761(1997)007[0737:HAOTGN]2.0.CO;2

[B84] Weber E. Engler C. Gruetzner R. Werner S. Marillonnet S. (2011). A modular cloning system for standardized assembly of multigene constructs. PLoS One 6. doi: 10.1371/journal.pone.0016765 PMC3041749 21364738

[B85] White J. F. Kingsley K. L. Zhang Q. Verma R. Obi N. Dvinskikh S.. (2019). Review: Endophytic microbes and their potential applications in crop management. Pest Manag Sci. 75, 2558–2565. doi: 10.1002/ps.5527 31228333 PMC6771842

[B86] Wilton R. Ahrendt A. J. Shinde S. Sholto-Douglas D. J. Johnson J. L. Brennan M. B.. (2018). A new suite of plasmid vectors for fluorescence-based imaging of root colonizing pseudomonads. Front. Plant Sci. 8. doi: 10.3389/fpls.2017.02242 PMC5799272 29449848

[B87] Xu P. Wang E. (2023). Diversity and regulation of symbiotic nitrogen fixation in plants. Curr. Biol. 33, R543–R559. doi: 10.1016/j.cub.2023.04.053 37279688

[B88] Young K. D. (2006). The selective value of bacterial shape. Microbiol. Mol. Biol. Rev. 70, 660. doi: 10.1128/MMBR.00001-06 16959965 PMC1594593

[B89] Zhang Y. Song H. Wang X. Zhou X. Zhang K. Chen X.. (2020). The roles of different types of trichomes in tomato resistance to cold, drought, whiteflies, and botrytis. Agronomy 10, 411. doi: 10.3390/AGRONOMY10030411

