# Supplementary material for: Gluconacetobacter diazotrophicus AZ0019 requires functional nifD gene for optimal plant growth promotion in tomato plants
Source: Front Plant Sci. 2024 Nov 22;15:1469676. doi: 10.3389/fpls.2024.1469676 (PMC11620874; doi:10.3389/fpls.2024.1469676)
Supplement: Supplementary file 1 [file DataSheet1.docx]

# **Supplementary Data**

Article title: *Gluconacetobacter diazotrophicus AZ0019* requires nitrogen fixing capabilities for optimal plant growth promotion

Authors: Michele Pallucchini, Martina Franchini, Enas M. El- Ballat, Nathalie Narraidoo, Benjamin Pointer-Gleadhill, Matthew J. Palframan, Christopher J. Hayes, David Dent, Edward C. Cocking, Michele Perazzolli, Rupert G. Fray and Phil J. Hill.

The following Supporting Information is available for this article:

**Fig S1:** *nifD* gene locus after disruption cassette integration.

**Fig S2:** Phenotype of unsuccessful seed coating application of Gd WT in glasshouse experiment.

**Fig S3:** Acetylene Reduction Assay (ARA) of Gd WT and Gd *nifD^-^* mutant.

**Fig S4:** Gd WT and Gd *nifD^-^* growth curve in hydroponic nutrient solution (NS) in the presence of 2 mM KNO_3_.

**Fig S5:** GUS staining on inoculated tomato plants grown hydroponically and divided in five anatomical zones as used for RT-qPCR analyses.

**Fig S6:** Colonisation of *A. thaliana* roots.

**Fig S7:** Colonisation of *A. thaliana* shoots.

**Methods S1:** Gd *nifD^-^* mutant construction

**Methods S2:** Acetylene Reduction Assay (ARA) protocol

**Table S1:** Bacterial strains list

**Table S2:** Primers used in this study

**Table S2:** Nutrient Solution for hydroponic systems

**Video S1:** *G. diazotrophicus* colonising tomato protoplasts

## **Supplementary Figures**

### Fig. S1


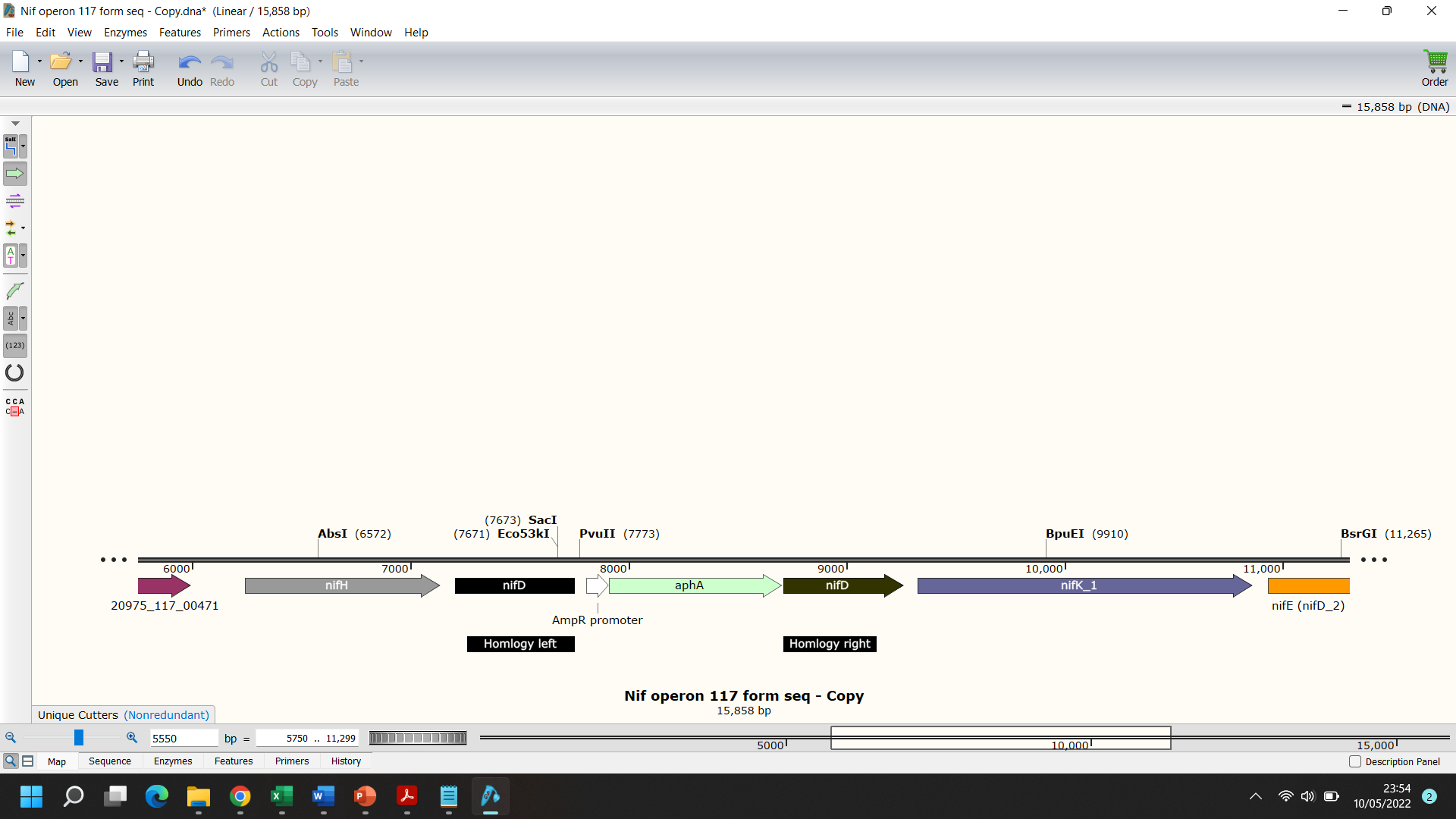

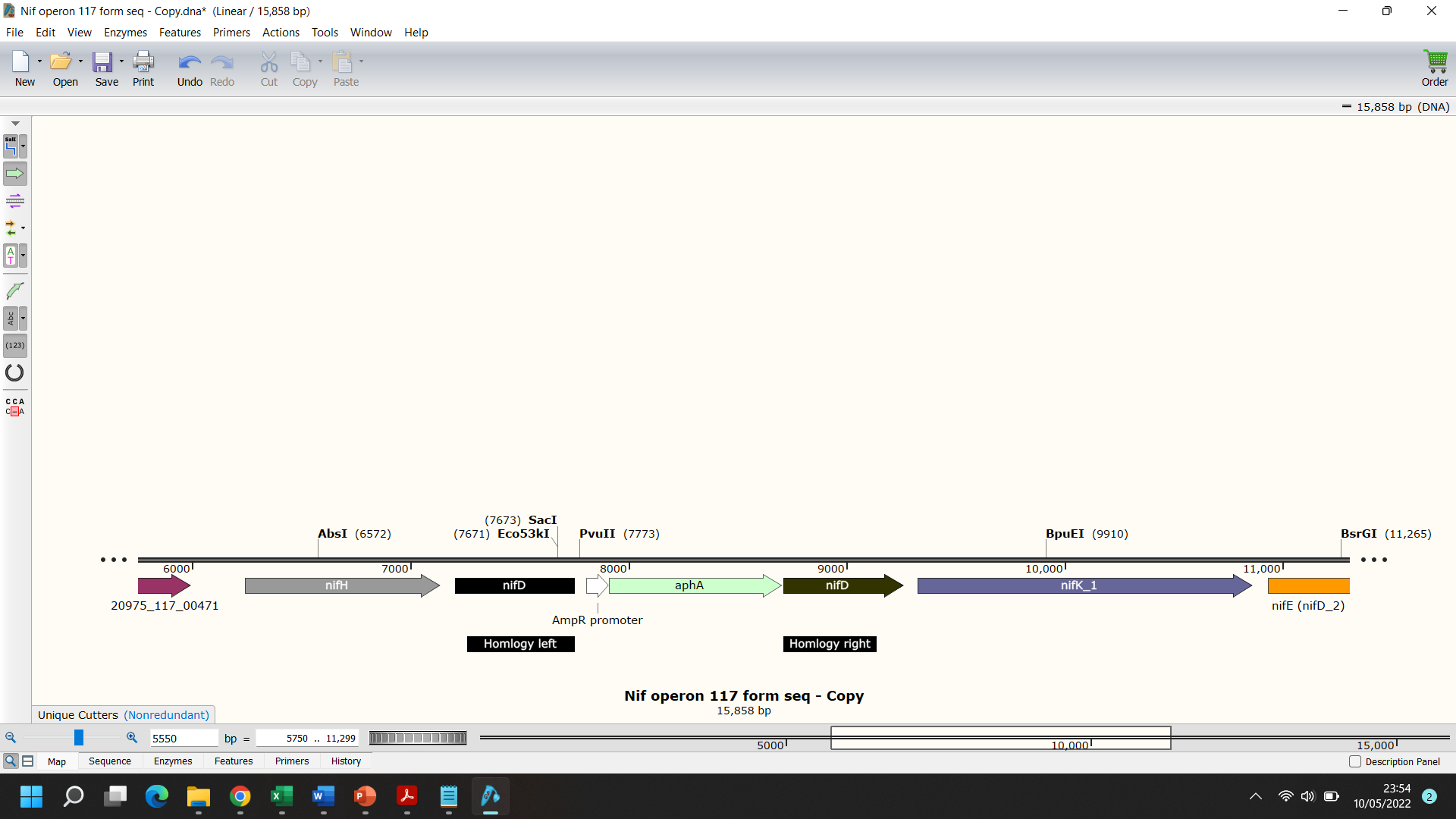

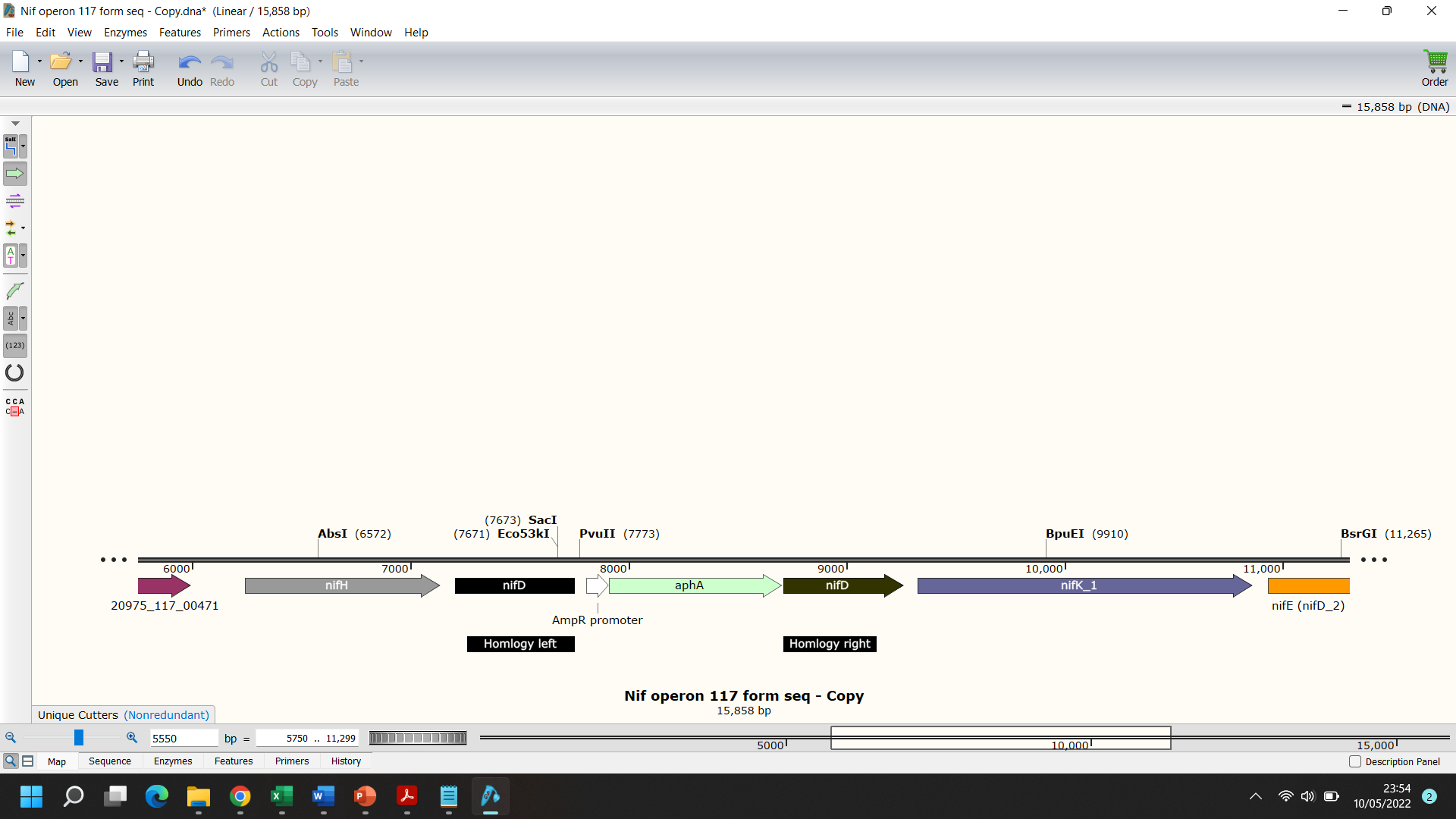


*Fig.S1. nifD gene locus after disruption cassette, visual representation as shown by annotated whole genome sequencing data.*

### Fig. S2


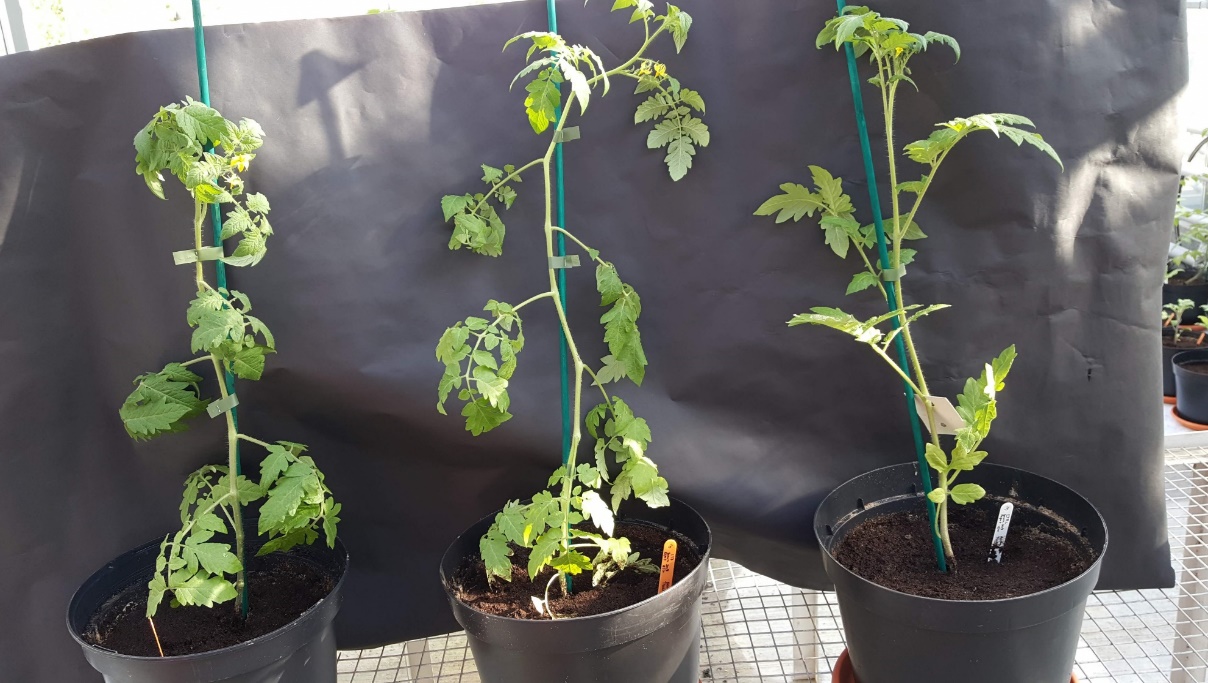

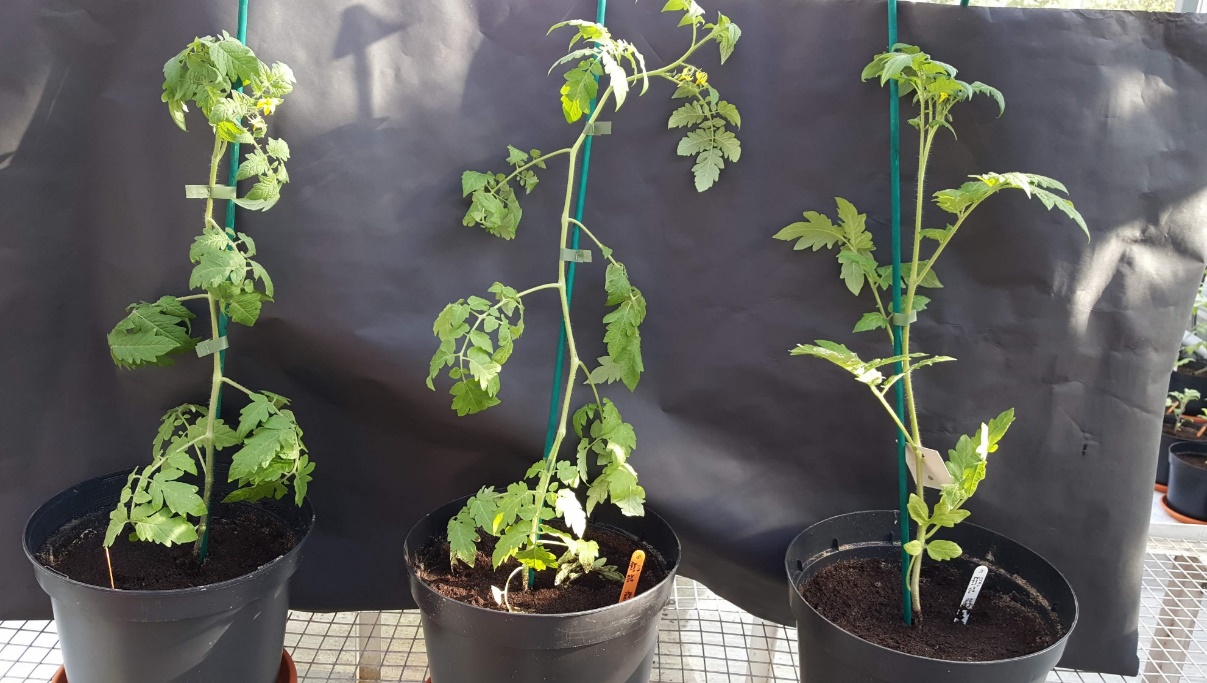


Ctrl

Gd WT

**(A)**

**(B)**

*Fig. S2. Phenotype of seed coating Gd WT application experiments in which the obvious PGP effect was reduced over time.  Mock-treated control plants (A) are compared to Gd WT 10^9^ CFU mL ^-1^ treated plants (B) at 2 months from sowing.*

### Fig. S3


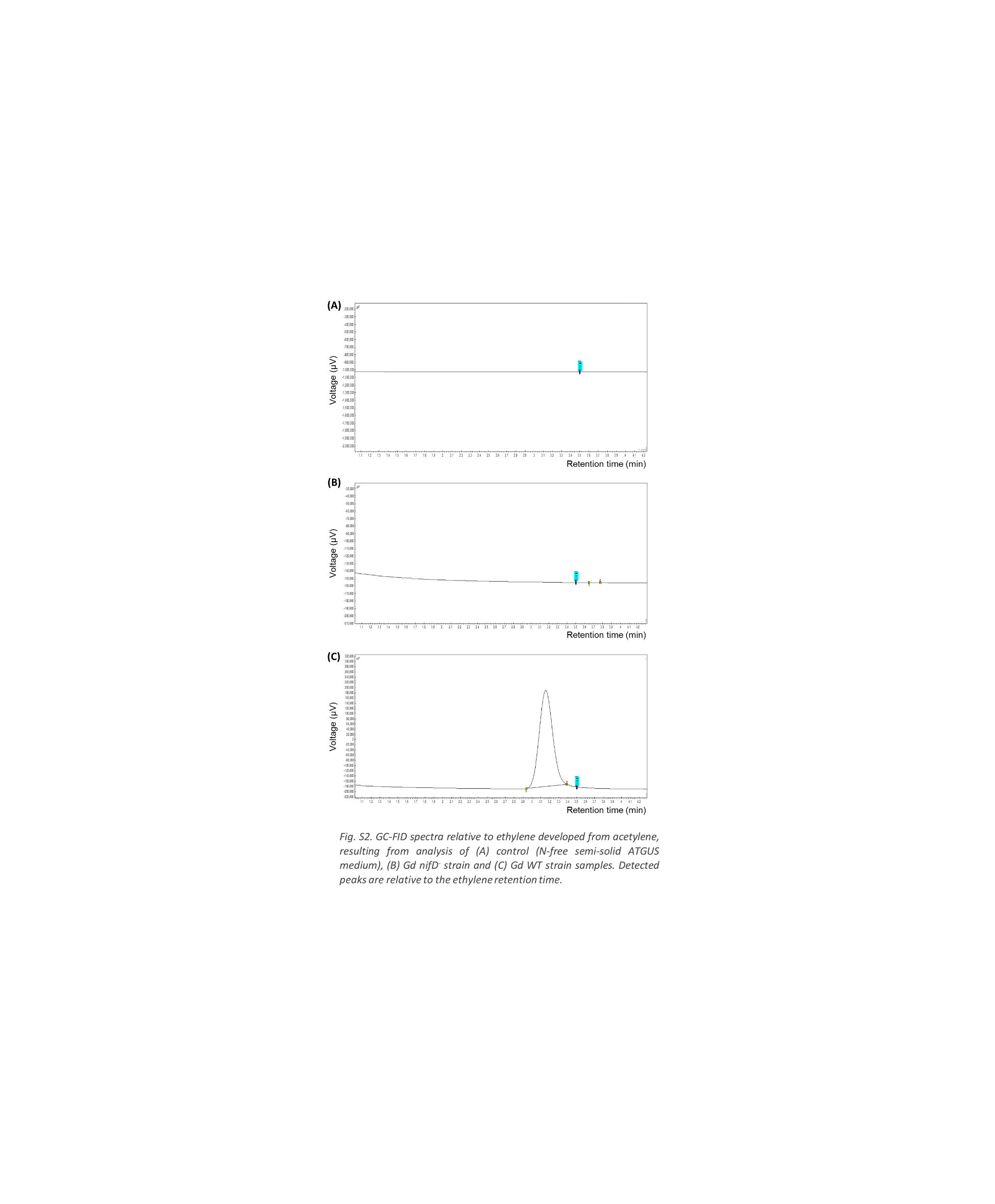


*Fig. S3. GC-FID spectra relative to ethylene developed from acetylene, resulting from analysis of (A) control (N-free semi-solid ATGUS medium), (B) Gd nifD^-^ strain and (C) Gd WT strain samples. Detected peaks are relative to the ethylene retention time.*

### Fig. S4

*Fig. S4.* *Gd WT and Gd nifD- growth curve in modified Hoagland nutrient solution (NS) in the presence of 2 mM KNO_3_. Incubation during repeated measurements at OD_600_ was performed at 25 °C in a BioTek Synergy™ microplate reader.*

### Fig. S5

*
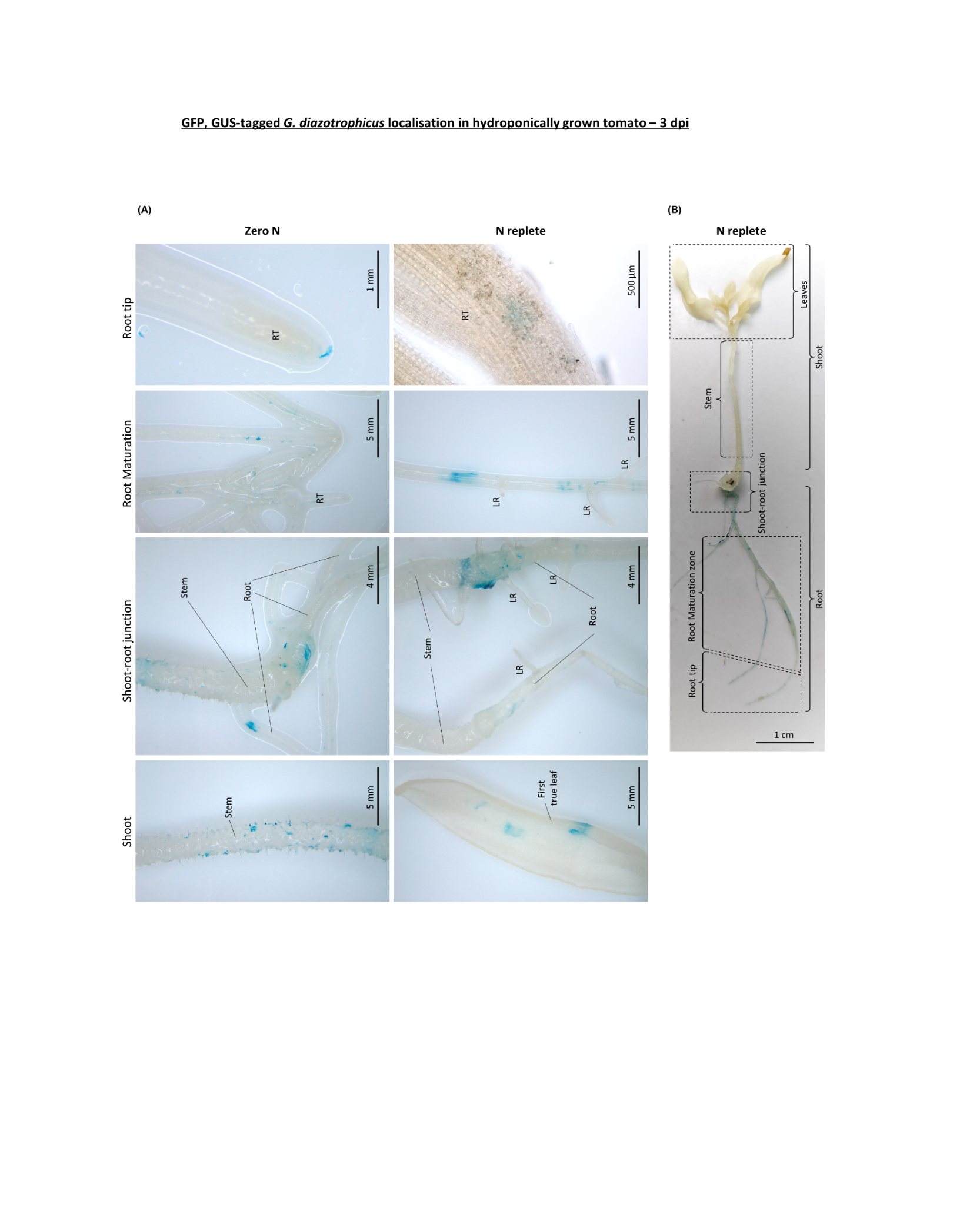
*

*Fig. S5. Tomato (cv. “MoneyMaker”) grown hydroponically in zero N or N replete (2mM KNO_3_) NS inoculated with GFP, GUS-tagged G. diazotrophicus* *at OD_600_ = 0.3 (10^7^ CFU mL^-1^), brightfield microscopy/stereoscopy. One day after the substitution of inoculated NS with fresh uninoculated NS (1 dpi), plants were rinsed in water to discard loosely adherent bacteria and subjected to X-Gluc staining overnight at 28 C°. (A) GUS signal (blue stains), indicating bacterial colonization, are shown on different plant tissues. (B) A representative GUS-stained whole plantlet is shown, illustrating the subdivision of the five anatomical zones analysed through qPCR (leaves, stem, root-shoot junction, root maturation zone and root tip, Fig. 4B) and the two anatomical regions (root and shoot) considered during gene expression analyses (Fig. 3) and MPN (Fig. 4A). LR = lateral roots; RT = root tip.*

### Fig. S6

*
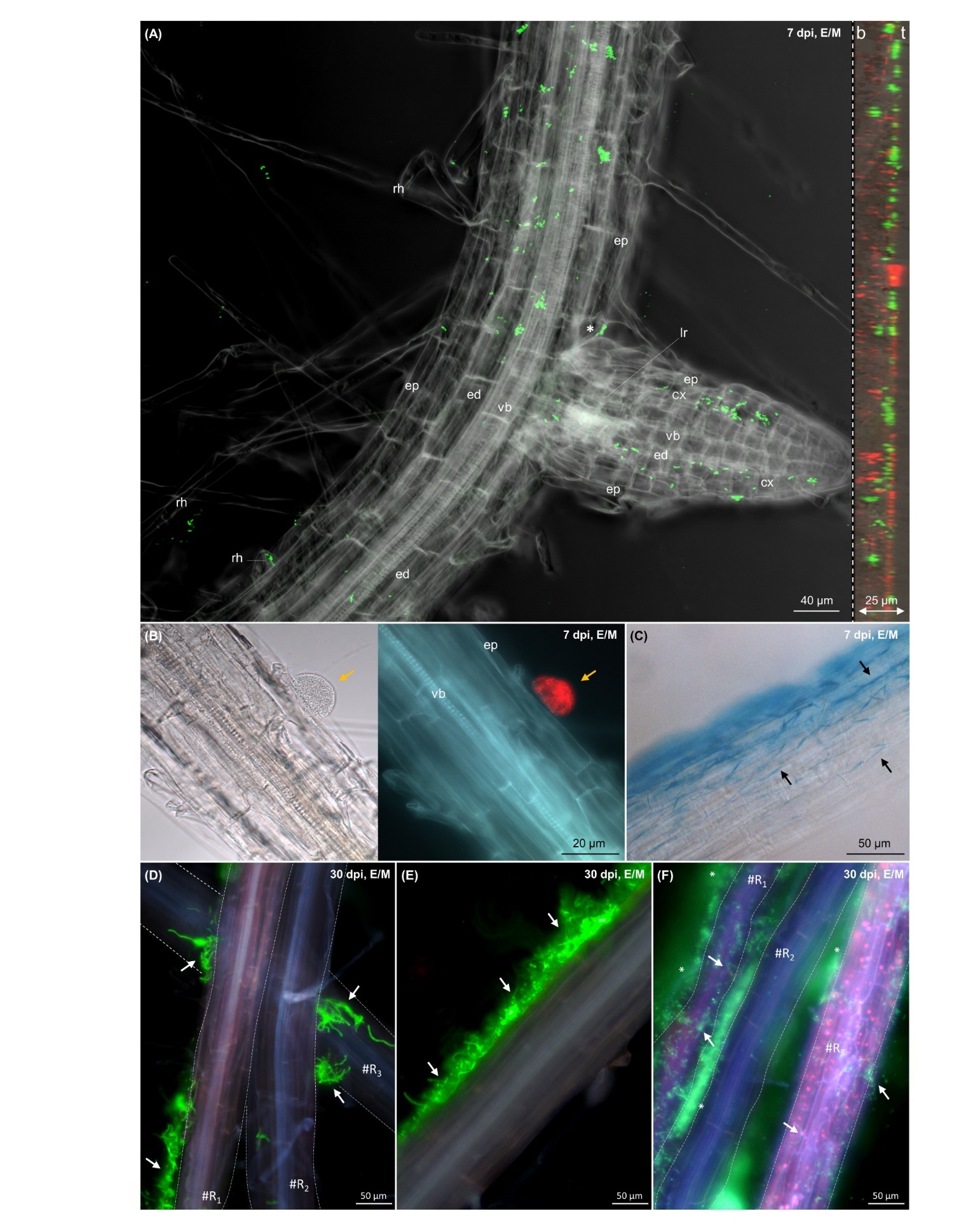
*

*Fig. S6. A. thaliana (Col-0) grown on NH_4_-free MS agar, inoculated with tagged G. diazotrophicus strains via vacuum infiltration (as described for Fig. 1 but under vacuum at 800 mbar). Fluorescence from GFPmut3* (A, D-F) and dsRed-Express2 (B) is visualized as green and red signals, respectively, through fluorescent microscopy. Bacterial β-glucuronidase activity (C) is visualized as blue signal through brightfield microscopy following X-Gluc staining. In the top right corner of each picture, the timepoint of observation and anatomical zone of the root are indicated (E = elongation; M = maturation. (A) Root cifferentiation/maturation zone colonization, maximum projection of a 25 images z-stack, for a total of 25 µm depth (confocal microscopy). On the right, the lateral view of the 3D model of the z-stack is included, with its right side corresponding to the top of the z-stack (root surface, t) and the left side corresponding to the z-stack bottom (root interior, b). Plastidial autofluorescence is shown in red as a reference for evaluating the penetration of G. diazotrophicus into the root. G. diazotrophicus clusters (green) exhibiting penetration within root tissues. (B) Spheroid biofilm (orange arrows) on the surface of A. thaliana roots (cyano), containing fluorescent coccoidal cells of dsRed-Express2-tagged G. diazotrophicus. (C) Elongated and filamentous G. diazotrophicus cells (black arrows) colonising intracellularly epidermal root cells. (D-F) Biofilm of concatenated, filamentous (white arrows) and rod-shaped bacteria colonising epiphytically the root epidermis. Nuclei autofluorescence is visualized as pink (F), while lignin autofluorescence is visualized as blue to purple. When multiple roots were present in the same image,* *they were demarcated individually with white dashed lines to facilitate precise identification and labelled as #R_n_.*

*ed = endodermis; ep = epidermis; rh = root hair; lr = lateral root; cx = cortex; vb = vascular bundle.*

### Fig. S7

*
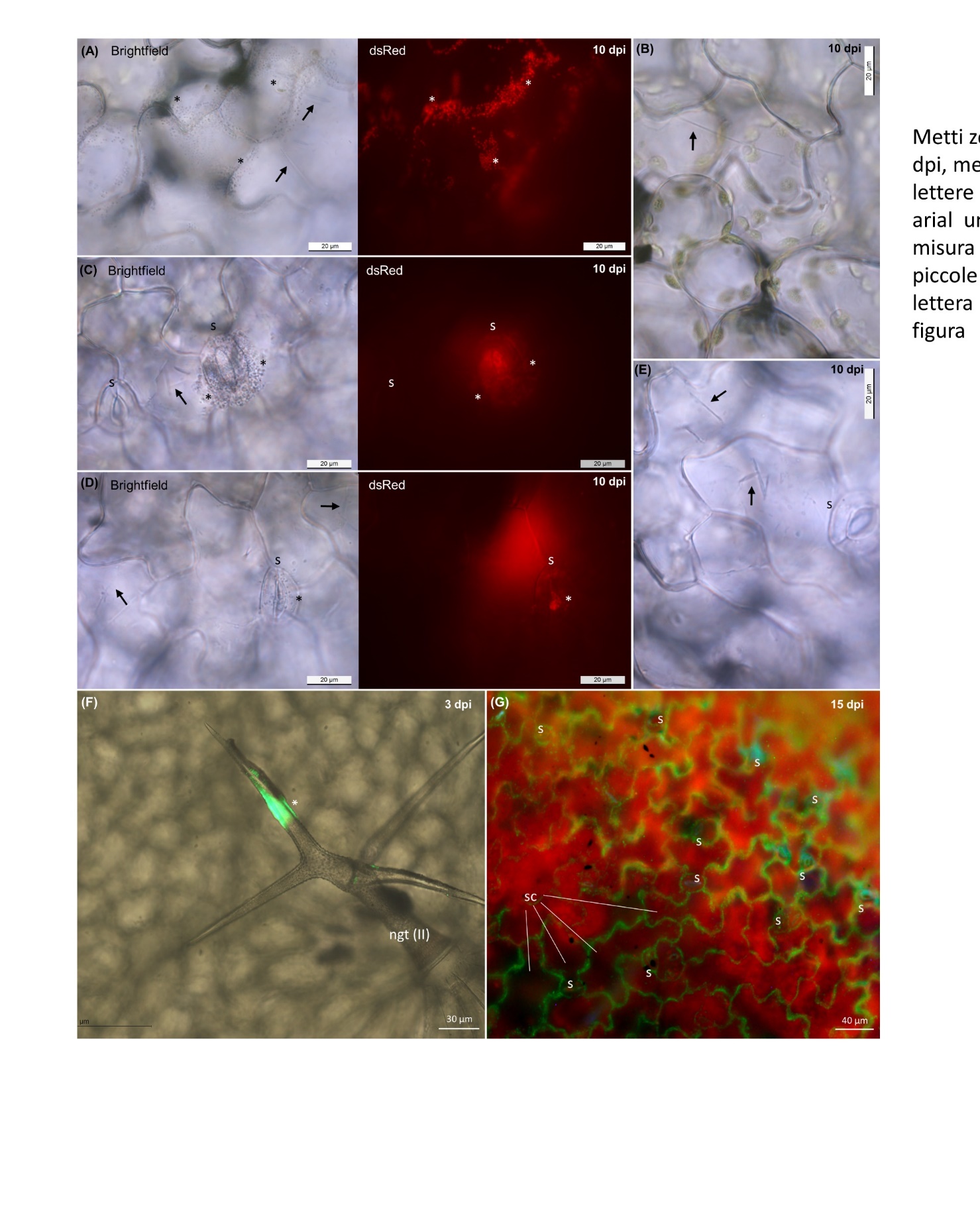
*

*Fig. S6. A. thaliana (Col-0) grown on NH_4_-free MS agar, inoculated with tagged G. diazotrophicus strains via foliar application. Fluorescence from GFPmut3* (F, G) and dsRed-Express2 A, C, D) is visualized as green and red signals, respectively, through fluorescent microscopy. In the top right corner of each picture, the timepoint of observation is indicated. (A) Coccoidal cells epiphytically colonising the leaf epidermis, mainly localising around cell wall junctions (white stars). (A-E) Non-fluorescent elongated or filamentous bacteria (black arrows) colonising leaf epidermis, often in proximity to colonized stomata. (C, D) Biofilm of coccoidal bacteria colonising and open stoma (C) and occupying a substomatal chamber of a semi-closed stoma (D). (F) Colonization of* *type II non-glandular trichome (white arrow). (G) Abaxial epidermis showing colonization of cell wall junctions, stomata and subsidiary cells. S = stoma; sc = subsidiary cell; ngt = non-glandular trichome.*

## **Supplementary Methods**

### **Methods S1. Gd nifD- mutant construction**

The Gd *nifD^-^* strain was produced by conjugation of Gd WT (AZ0019) with the λ*pir* *E.coli β2163* carrying a *nifD^-^* disruption cassette assembled in the pSW23T plasmid (R6K γ *ori*). The cassette was designed by inserting a kanamycin resistance (Kan^R^) between the 5’ and 3’ homology *nifD* ends (492 and 424 bp, respectively). The conjugation was performed on PDA medium supplemented with diaminopimelic acid (30 mM DAP) and incubated for 48 hrs at 28°C. Gd Transformants which underwent a double recombination event were selected on ATGUS medium without DAP and screened through colony PCR. The strain genotype was confirmed through whole genome sequencing (Fig S1), combining Illumina and NanoPore technologies (Deep Seq Team, D106, QMC, University of Nottingham).

### **Methods S2. Acetylene Reduction Assay (ARA) protocol**

Previously autoclaved 30 mL glass vials containing semi-solid ATGUS N-Free medium (0.5% agar) were inoculated with 10 µl MilliQ Water (Control) or 10 µl of bacterial liquid cultures of Gd WT or Gd *nifD*^-^ at the same optical density. Three biological replicates were set per each treatment. Incubation was carried out for 72 hrs at 28 °C, after which a pellicle of comparable diameter and density had appeared on the surface of the semisolid medium (with the *nifD*^-^ strain relying for its growth on carryover N from the pre-inoculation culture). Vials were stoppered with sterile, gas-tight, rubber stoppers with a pierceable septum and 1 mL of acetylene was added to the headspace with a 1 mL sterile syringe. Incubation was carried out for a further 72 hrs at 28 °C. Acetylene gas was developed from CaC_2_ granules mixed with sterile distilled water. Calculations for the required amount of acetylene to be developed from CaC_2_ were referred to the proportion recommended by (Castle, 2010) for Acetylene Reduction Assay. Analysis of the headspace was performed with a Gas Chromatographer (GC) equipped with a Flame Ionisation Detector (FID) set at 60 °C with the injector at 110 °C, with 3 mL min^−1^ flow rate of carrier gas. 1 mL of the headspace was manually injected with 1 mL sterile syringes equipped with 23- or 25-gauge needles into the GC. The presence of peaks between 3 and 3.2 minutes retention time, corresponding to ethylene, was evaluated (Fig S2). The analysis detected a conversion of acetylene (C_2_H_2_) to ethylene (C_2_H_4_) in the Gd WT strain (Fig. S 2c), while no ethylene production was observed in the headspace of Gd *nifD-* or water inoculated vials (Fig. S 2b and S 2a, respectively). This result confirmed the inactivation of the nitrogenase complex in the Gd *nifD^-^* strain.

Ethylene standards were employed as reference to quantify the amount of acetylene reduced. The average concentration obtained for the three replicates relative to Gd WT strain is 222.06 ± 141.76 ppm.

## **Supplementary Tables**

### *Table S 1. Bacterial strains characteristics and sources.*

| Bacterial strain | Description and relevant characteristics | Antibiotic working concentration | Source |
| --- | --- | --- | --- |
| *G. diazotrophicus* AZ0019 |  |  | Azotic Technologies Ltd. |
| *G. diazotrophicus nifD^-^* | *nifD*^-^ [Kan^R^] | Kan 50 µg mL^-1^ | R. Fray Lab, UoN |
| *E. coli β2163* | (F-) RP4-2-Tc::*Mu* *ΔdapA*::(*erm-pir*) [Kan^R^; Em^R^] | DAP 300 µM  Kan 50 µg mL^-1^ | R. Fray Lab, UoN  (Demarre et al., 2005) |
| *G. diazotrophicus* GUS, GFP tagged | pBBR1MCS5 [Gent^R^,Cm^R^] for constitutive *gfpmut3*;* pRGS561 [Spe^R^; St^R^; Kan^R^] for constitutive *gusA* expression | Kan 50 µg mL^-1^  Cm 25 µg mL^-1^  Gent 25 µg mL^-1^  Strept/Spect 50 µg mL^-1^ | R. Fray Lab, UoN |
| *G. diazotrophicus* dsRed tagged | pICH47751_DsRed for constitutive *dsRed-Express2* expression | Kan 50 µg mL^-1^ | R. Fray Lab, UoN |

### *Table S 2. Primers relevant to this study.*

| 11 F | 5’-TCGAGCAGCCGTTTCATCCA-3’ |
| --- | --- |
| 11 R | 5’-TCAGGGCAATCACTAGCCGG-3’ |
| 12 F | 5’-TGATGCGCTTGTTCGTGACG-3’ |
| 12 R | 5’-CGTTCGCCCTTGTCGTCATG-3’ |
| Rho Fw | 5’-GCAACTCGGAACTCATCCTC-3’ |
| Rho Rv | 5’-GGACCCACATCTTCGACAGT-3’ |
| nifD Fw | 5’-AGTTCACGTCCGATTTCCAG-3’ |
| nifD Rv | 5’-CGGAAAGAGCTCATCGATTT-3’ |
| ard2 Fw | 5’-TGTTCATCAGTGTGCTAGTG-3’ |
| ard2 Rv | 5’-GCTGTCCTTCCTTCTGAATC-3’ |

### *Table S 3. Nutrient Solution recipe – zero nitrogen*

| Compound | Desired compound concentration  (1xNS) |
| --- | --- |
|  | **mg L^-1^** |
| K_2_SO_4_ | 261.39 |
| MgSO_4_ 7H_2_O | 369.72 |
| KH_2_PO_4_ | 136.09 |
| NaCl | 29.23 |
| Ca(H_2_PO_4_)_2_ H_2_O | 116.03 |
| CaCl_2_ 2H_2_0 | 294.02 |
| FeSO_4_ 7H_2_0 & EDTA 2Na 2H_2_0 | 13.005 |
| CuSO_4_ 5H_2_O | 0.5 |
| ZnSO_4_ 7H_2_O | 3 |
| MnSO_4_ 4H_2_O | 2 |
| H_3_BO_3_ | 4 |
| Na_2_MoO_4_ 2H_2_O | 0.5 |

## **References**

Castle, S. (2010). Acetylene Reduction Assay (ARA): Measuring Nitrogenase Activity. *Adapted from Hawkes Lab*.

Demarre, G., Guérout, A. M., Matsumoto-Mashimo, C., Rowe-Magnus, D. A., Marlière, P., & Mazel, D. (2005). A new family of mobilizable suicide plasmids based on broad host range R388 plasmid (IncW) and RP4 plasmid (IncPα) conjugative machineries and their cognate Escherichia coli host strains. *Research in Microbiology*, *156*(2), 245–255. https://doi.org/10.1016/J.RESMIC.2004.09.007
